# A Current Overview of the Biological and Cellular Effects of Nanosilver

**DOI:** 10.3390/ijms19072030

**Published:** 2018-07-12

**Authors:** Shana J. Cameron, Farah Hosseinian, William G. Willmore

**Affiliations:** 1 Department of Chemistry, Carleton University, 1125 Colonel by Drive, Ottawa, ON K1S 5B6, Canada; shana.cameron@carleton.ca (S.J.C.); farah.hosseinian@carleton.ca (F.H.); 2 Institute of Biochemistry, Carleton University, 1125 Colonel by Drive, Ottawa, ON K1S 5B6, Canada; 3 Department of Biology, Carleton University, 1125 Colonel by Drive, Ottawa, ON K1S 5B6, Canada

**Keywords:** silver nanoparticles, nanosilver, endocytosis, oxidative stress, reactive oxygen species, inflammation, wound healing, hypoxia, mitochondria, endoplasmic reticulum stress, unfolded protein response, autophagy, apoptosis, angiogenesis, epigenetics, genotoxicity, cancer, anti-cancer

## Abstract

Nanosilver plays an important role in nanoscience and nanotechnology, and is becoming increasingly used for applications in nanomedicine. Nanosilver ranges from 1 to 100 nanometers in diameter. Smaller particles more readily enter cells and interact with the cellular components. The exposure dose, particle size, coating, and aggregation state of the nanosilver, as well as the cell type or organism on which it is tested, are all large determining factors on the effect and potential toxicity of nanosilver. A high exposure dose to nanosilver alters the cellular stress responses and initiates cascades of signalling that can eventually trigger organelle autophagy and apoptosis. This review summarizes the current knowledge of the effects of nanosilver on cellular metabolic function and response to stress. Both the causative effects of nanosilver on oxidative stress, endoplasmic reticulum stress, and hypoxic stress—as well as the effects of nanosilver on the responses to such stresses—are outlined. The interactions and effects of nanosilver on cellular uptake, oxidative stress (reactive oxygen species), inflammation, hypoxic response, mitochondrial function, endoplasmic reticulum (ER) function and the unfolded protein response, autophagy and apoptosis, angiogenesis, epigenetics, genotoxicity, and cancer development and tumorigenesis—as well as other pathway alterations—are examined in this review.

## 1. Introduction

Nanosilver is made up of extremely small particles of silver, with lengths of 1–100 nm in at least one dimension [1]. Commercially, it is often named colloidal silver or silver nanoparticles. Nanosilver is well recognized to have antimicrobial, antifungal, and antiviral properties, and for these reasons it is widely used today in many consumer products such as food packaging, sports clothing, electronics, cosmetics, medical devices, and bandages [2,3,4,5,6]. Nanosilver is used as an effective treatment against both Gram-negative and Gram-positive bacteria [7,8], as well as against human immunodeficiency virus (HIV) [9,10]. Nanosilver also has antifilarial activity [11,12]. The number of applications of nanosilver, and its current usage in commercial products, means that unregulated environmental contamination by nanosilver and human exposure to nanosilver is now a reality. A cause for concern is that nanosilver surface oxidation releases Ag^+^ ions which are known to be toxic [1]. In fact, the smaller the nanosilver particle, the higher the surface area to volume ratio, and the more Ag^+^ ions are released. The antimicrobial properties of nanosilver are due to this release of Ag^+^ ions [5] as well as due to nanosilver-specific effects, such as inhibition of transcription by ribonucleic acid (RNA) polymerase [13]. The release of Ag^+^ ions inside cells from nanosilver that entered in its nanoparticle form has been sometimes referred to as the “Trojan horse” effect [13,14,15,16]. However, the effects due to the released Ag^+^ ions versus the effects due to the non-ionic nanosilver particle in cells is still under investigation, and varies depending on the experimental conditions [1]. Manshian et al. [17] measured the amount of Ag^+^ ions released from nanosilver solutions by inductively coupled plasma-mass spectrometry (ICP-MS), treated primary human umbilical vein endothelial cells (HUVEC) and murine C17.2 neural progenitor cells with silver nitrate (AgNO_3_) at the appropriate concentrations corresponding to the nanosilver treatments, and found only slight toxicity directly from the Ag^+^ ions. Lin et al. [18] used a filter assay and ICP-MS to determine the amount of Ag^+^ ions released from a solution of polyvinylpyrrolidone (PVP)-coated nanosilver after 24 h, and found that level of Ag^+^ ions to have no appreciable effect on the cells. The release of Ag^+^ ions from PVP-coated nanosilver (25 nm) was found to be 9.3% in cell culture media, with this concentration of AgNO_3_ not decreasing the viability of human embryonic kidney (HEK 293T) cells [14]. A release of Ag^+^ ions of 17.9% and 10.3% has also been reported in nanosilver treated mouse erythroleukemia (MEL) and human liver carcinoma (HepG2) cells, respectively [13]. In immortalized murine bone-marrow derived pro-B (Ba/F3) cells, 21.6% of the nanosilver was released as Ag^+^ ions, while no Ag^+^ ions were detected in either deionized water or cell culture media with the same concentration of nanosilver [19]. The size of the nanosilver particle affects the quantity of Ag^+^ ions that are released, and 10 nm citrate-coated nanosilver was found to release 22% as Ag^+^ ions, while 40 nm citrate-coated nanosilver released only 11% as Ag^+^ ions after 24 h in the cell culture media [20]. Silver ion selective electrodes have also been used to measure the amount of Ag^+^ ions released from nanosilver [13,21]; and these studies indicate that the nanosilver itself, and not just the released Ag^+^ ions, affect the cellular processes. In addition, silver may be taken up and localized in the cell differently depending on whether it is in its nanoparticle or ionic form, resulting in specific nanoparticle effects. Various factors can affect the quantity of Ag^+^ ions released such as the size of the nanosilver, the pH of the environment it is in, the surface coating, or the formation of a protein cornea around the nanosilver [17,22,23,24]. In cells, nanosilver undergoes transformation from the elemental silver (Ag^0^), to Ag^+^ ions, to silver oxide species (Ag–O–), and finally to silver sulfide species (Ag–S–) upon binding to thiols [25]. In the environment, nanosilver undergoes sulfidation reactions to Ag_2_S, greatly reducing its potential toxicity [26,27,28]. Lesser amounts of nanosilver are transformed into organic sulfide thiol complexes, AgNO_3_, Ag–lactate, silver chloride (AgCl), or Ag_2_O, with only minor amounts being released as free aqueous Ag^+^ ions [29,30,31].

Nanosilver can be absorbed through inhalation (e.g., shoe sprays or during industrial manufacturing), oral ingestion (e.g., from food packaging or taken medicinally), skin contact (e.g., from bandages, cosmetics, or clothing), and injection (e.g., medicinal) [3,24,32]. Once in the body, nanosilver is transported to the liver via the portal vein. The nanosilver is released into the blood stream where it can then bind to blood plasma proteins and blood cells and be distributed to all the organs in the body [32]. Nanosilver is able to cross the blood brain barrier as well as the placental barrier [3,33,34]. A 28 day oral exposure study by Van der Zande et al. [35] of male Sprague Dawley rats to nanosilver (17.7 nm, polyoxyethylene glycerol trioleate/Tween 20 stabilized; or 12.1 nm, PVP-coated) indicated that the highest levels of nanosilver were found in the liver and spleen, with lower levels being found in the testis, kidney, brain, and lungs. Inhaled nanosilver may also reach the brain through the olfactory bulb [36]. A gender-specific difference in nanosilver accumulation was seen in a 90-day oral exposure study with ~60 nm nanosilver, where it was found that female Fischer 344 rats accumulated twice the amount of silver in their kidneys as did the male rats [37,38,39]. In terms of intracellular distribution, a related study examining the effects of AgCl fed to albino rats over one month resulted in Ag^+^ ions mainly in the mitochondria of the liver cells, while rats fed AgCl over six months resulted in Ag^+^ ions mainly located in the cytosol of the liver cells [40]. Exposure to large amounts of nanosilver over a long period of time can result in a condition known as argyria, where silver is deposited in the skin microvessels; or argyrosis, where silver is deposited in the eyes, causing a permanent bluish discolouration [1,41,42]. Excretion of nanosilver (17.7 nm, polyoxyethylene glycerol trioleate/Tween 20 stabilized; or 12.1 nm, PVP-coated) from orally treated male Sprague Dawley rats mainly occurs through the feces (>99%), with trace amounts of the nanosilver being excreted in the urine [35].

The physical characteristics of nanosilver such as size, shape, coating, and aggregation state are very important in its interactions and effects on living organisms [24]. Larger sized nanosilver (100 nm) may not enter the cell, and instead may exert indirect receptor-mediated signalling effects such as through serine/threonine protein kinase (PAK), mitogen-activated protein kinase (MAPK), and protein phosphatase 2A (PP2A) [43]. Smaller nanosilver particles can enter the cells, release Ag^+^ ions, interact with the various biomolecules, and may bind to sulfur containing proteins and peptides such as glutathione (GSH), thioredoxin (TXN), thioredoxin peroxidase, and superoxide dismutase (SOD) through their sulfhydryl groups [1,5,24,43].

The size of the nanoparticles reported in this review are the size of the metallic core, generally as indicated by transmission electron microscopy (TEM) imaging in the studies, or failing that, the size specified by the supplier. This size does not include the surface coating or hydration shell around the nanosilver particle. Additionally, nanosilver is sometimes found to aggregate in the cell culture media depending on the surface coating of the nanosilver used or the handling method [44], making the size even larger. PVP is one of the common nanosilver coatings used in experiments to stabilize the nanosilver and prevent aggregation [45]. The effects of PVP itself has been tested at the appropriate experimental concentrations on cells and found to not cause the effects that are observed when the cells are treated with PVP-coated nanosilver [45,46,47]. Citrate is another common stabilizer, and on its own did not decrease the life span of *Drosophila melanogaster* [21]. The dosage of the nanosilver is also very important in terms of the cellular effects and toxicity. Many studies use a high and toxic concentration in their experiments however, lower non-toxic doses are more relevant to the actual environmental exposure levels [21]. A hormetic effect has been observed with lower doses triggering cell-survival pathways and somewhat protecting the cells against subsequent higher dose treatment which leads to cell death [24,48,49].

The use of controls in nanosilver studies is important for determining the cause of the observed effects. AgNO_3_ is most commonly used as an Ag^+^ ion control [50]; however, silver acetate (C_2_H_3_AgO_2_) [51,52] or silver carbonate (Ag_2_CO_3_) have also been used [53]. If the Ag^+^ ion control is used at the same concentration as the nanosilver treatment dose, the AgNO_3_ will be much more toxic since there are many more silver ions present than in the nanosilver solution [21,54]. In order to treat cells with a relevant concentration of Ag^+^ ions for the Ag^+^ ion control: (1) ICP-MS may be performed on the nanosilver solution to determine the concentration of Ag^+^ ions that are released [13,17,18,54]; (2) viability assays may be done to determine the treatment concentrations for both the Ag^+^ ion control and nanosilver that gives the same percentage of cell viability [55]; or (3) the nanosilver particles can be incubated in media for an experimentally relevant time, removed by centrifugation, and the cells then treated with the remaining media containing any released Ag^+^ ions [43,56]. A nanoparticle control such as cerium (Ce) nanoparticles [18,50] or polystyrene nanoparticles [53] may also be used, although this control is less common in nanosilver studies.

**Figure 1:**
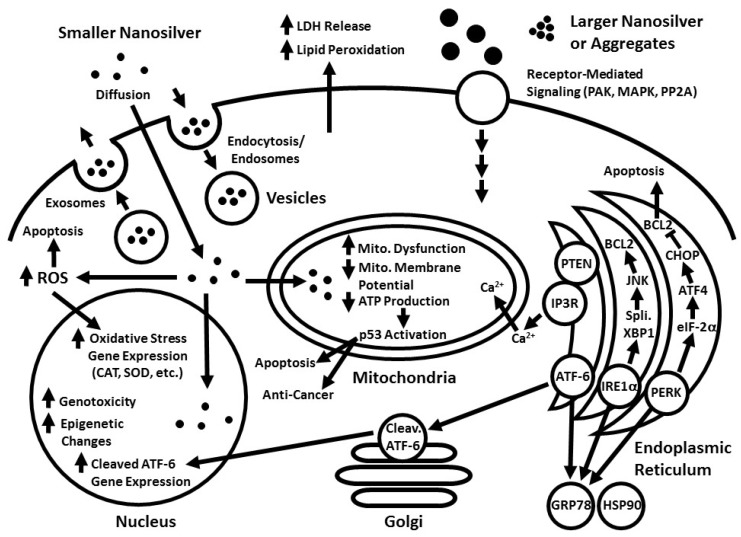
Effects of silver nanoparticles on the cell stress response pathways. Smaller sized nanosilver (~10 nm diameter) enters the cell either through being taken up into endosomes/lysosomes by endocytosis or through simple diffusion across the cell membrane (potentially due to induced lipid peroxidation and disruption of the plasma membrane). Larger sized nanosilver or large aggregates of nanosilver cannot enter the cell by these means, but can instead activate various receptor-mediated signalling mechanisms, such as through PAK, MAPK, and PP2A. Increased lipid peroxidation causes increased LDH release from the cell due to cell membrane damage. Nanosilver treatment results in an increase in reactive oxygen species (ROS), and the extrinsic apoptotic pathway may be induced. The levels of reduced glutathione (GSH), superoxide dismutase (SOD), and catalase (CAT) are affected and an increase in oxidative stress response gene expression occurs. In the nucleus, an increase may occur in genotoxicity (DNA damage, DNA base oxidation, DNA adducts, DNA strand breaks, and chromosomal aberrations) and epigenetic changes (DNA methylation, various histone tail modifications, and changes in non-coding RNA expression), potentially in a transient manner. Mitochondrial dysfunction, decreased mitochondrial membrane potential, decreased ATP production, and mitochondrial-mediated intrinsic apoptosis may also occur. As well, nanosilver treatment increases the protein and gene expression levels of p53, leading to anti-cancer effects. High dose nanosilver treatment disrupts endoplasmic reticulum (ER) homeostasis and induces the ER stress response through activated PERK, ATF-6, and IRE-1α, and their respective pathways. Contact between the ER and the mitochondria increases with nanosilver treatment, and increased transfer of calcium from the ER to the mitochondria occurs, resulting in increased calcium levels in the mitochondria.

This review examines how nanosilver of various sizes and coatings enters or interacts with cells, and the resulting biological and cellular effects (Figure 1).

## 2. Cellular Uptake and Localization of Nanosilver

Nanosilver is mainly taken up into cells through endocytosis into vesicles, although diffusion of the nanosilver across the cell membrane into the cytoplasm may also occur [54,57,58,59,60]. In endocytosis, the material is taken up into early endosomes formed from the cell membrane. These develop into late endosomes and then into lysosomes, which have a lower internal pH [52]. The acidic environment in the lysosomes increases the release of Ag^+^ ions from the nanosilver [24]. Nanosilver particles may also be able to diffuse across the membrane through induced lipid peroxidation and disruption of the plasma membrane [5,24]. Additionally, nanosilver and Ag^+^ ions have been known to interact with copper transport channels and may be taken up through these [21,24,61].

In a DNA microarray study, non-toxic treatment of HepG2 cells with 20 nm citrate-coated nanosilver for 4 h increased the expression of genes for both clathrin-dependent and clathrin-independent endocytosis [58]. Human mesenchymal stem cells (hMSC) treated with non-toxic levels of 50 nm PVP-coated nanosilver took up the nanosilver through clathrin-dependent endocytosis and macropinocytosis. Nanosilver aggregates accumulated inside late endosomes or lysosomes, but were not seen in the nucleus, ER, or Golgi apparatus using fluorescence and light microscopy [52]. Similar results were found in normal human lung fibroblast (IMR-90) cells treated with 6–20 nm nanosilver, where the nanosilver was taken up through clathrin-dependent endocytosis and micropinocytosis into endosomes and the nucleus, and then removed through exocytosis [62]. TEM images showed 10, 50, and 100 nm PVP-coated nanosilver to be contained within single or double membrane vesicles in HepG2 cells, with some of the 100 nm particles breaking down into smaller particles. Energy dispersive X-ray spectroscopy (EDS) further confirmed that silver was in the vesicles [63]. TEM images of human acute monocytic leukemia cells (THP-1) treated with cytotoxic levels of 20 nm nanosilver showed that the nanosilver was contained in endosomes or lysosomes within the cells, but not in the nucleus or mitochondria. Nanosilver contained in these vesicles was then removed from the cells through exocytosis [25]. Similar results were also seen in Chinese hamster ovary subclone K1 (CHO-K1) cells and NIH 3T3 mouse embryonic fibroblast cells, where nanosilver was observed to be contained in endosomes or lysosomes, but not inside the nucleus, mitochondria, or Golgi apparatus [64,65], and non-toxic nanosilver (citrate-coated, 15.26 nm) treatment of U251 glioblastoma cells resulted in aggregates contained in endosomes [66]. In vivo examination of the livers of male Sprague Dawley rats treated with nanosilver (PVP-coated, 22.32 nm) via intraperitoneal injection revealed that the nanosilver was deposited in endosomes and lysosomes in Kupffer cells, and was generally found in the liver cells closest to the blood vessels [67]. Some studies report the presence of nanosilver inside the mitochondria and nucleus. Nanosilver was found inside the mitochondria in human blood monocytes treated with 28 nm nanosilver [59]. Aggregates of nanosilver were observed to be free in the cytoplasm, contained in vesicles, and in the nucleus in hMSC cells treated with 46 nm nanosilver [57]. Nanosilver was taken up into the cytoplasm and nucleus in male somatic Leydig (TM3) cells and male somatic Sertoli (TM4) cells treated with 10 nm nanosilver at the concentration that results in 50% cell death, that is, the effective concentration that results in 50% of the maximal effect (EC_50_) [68]. Finally, ICP-MS indicated the presence of silver in the cytosol and nucleus, with only a low amount of silver being detected in the membrane fraction in MEL cells treated with 25 nm PVP-coated nanosilver [13].

The amount of nanosilver internalized by cells depends on the size, shape, surface coating, and surface charge of the nanosilver [50,69]. Smaller sized nanosilver has been repeatedly reported to elicit greater cellular effects than larger nanosilver particles due to both increased cellular uptake and increased intracellular interactions. HepG2 cells treated with 10 and 75 nm, PVP and citrate-coated nanosilver, internalized the most nanosilver when treated with the citrate-coated nanosilver, followed by PVP-coated nanosilver, and took up the least when treated with the silver nitrate control. The smaller 10 nm nanosilver elicited a stronger oxidative stress pathway response than the larger 75 nm nanosilver [50]. In rat N27 neuronal cells treated with non-toxic levels of 10 and 75 nm, PVP and citrate coated nanosilver, the PVP-coated nanosilver affected gene expression more than the citrate-coated nanosilver. Additionally, the smaller 10 nm PVP-coated nanosilver elicited stronger nuclear factor (erythroid-derived 2)-like 2 (Nrf2) transcription factor and antioxidant response element (ARE) related gene activation, while than the larger 75 nm PVP-coated nanosilver activated more genes related to mitochondrial dysfunction, DNA damage, and kidney damage [69]. Smaller 10 and 25 nm PVP-coated nanosilver inhibited globin mRNA expression in MEL cells more than larger 40 and 110 nm nanosilver, and spherical nanosilver had a stronger effect than plate-like nanosilver [13].

Cell type and function is also a large determining factor for nanosilver absorption. For example, phagocytic mouse BV2 microglia cells took up nanosilver of all the sizes and coatings tested, while non-phagocytic N27 neuronal cells took up very little nanosilver [69].

## 3. Nanosilver and Oxidative Stress (Reactive Oxygen Species)

Reactive oxygen species (ROS) are any form of oxygen with an unpaired electron in its outer electron orbital, such as superoxide (•O_2_^−^), hydrogen peroxide (H_2_O_2_), the hydroxyl radical (•OH), singlet oxygen (^1^O_2_), alkoxy radicals (RO•), peroxy radicals (ROO•), hydochlorous acid, hypobromous acid, and others [70]. ROS are mainly produced in the mitochondria in cells. During oxidative phosphorylation, some electrons may escape and bind to O_2_ forming •O_2_^−^, which may be converted to other ROS such as H_2_O_2_ and •OH. Inhibition of oxidative phosphorylation may result in increased formation of •O_2_^−^, as well as decreased ATP production [62]. Malondialdehyde (MDA) is produced as a result of lipid peroxidation and increased MDA is an indication of oxidative stress [71,72]. Superoxide dismutase (SOD) neutralizes •O_2_^−^ by converting it to oxygen (O_2_) and H_2_O_2_, and H_2_O_2_ is then converted to water (H_2_O) and O_2_ by catalase (CAT). GSH is the major antioxidant molecule produced in cells [73]. Intracellular ROS is of interest since low amounts of ROS are important signalling molecules; however, large amounts can deplete the levels of GSH, activate the cellular antioxidant response, cause cellular damage such as the oxidation of proteins and DNA [62,74], cause mitochondrial damage, and ultimately lead to cell death if the cell cannot respond sufficiently [1,75,76]. It has been generally thought that cellular nanosilver toxicity is mainly due to the production of ROS in the cell [1,19,75,76], however, some studies report no change or a decrease in ROS with nanosilver treatment. Many different factors may be contributing to these results, including the methods used for detecting ROS, the exact ROS being measured, the relative sensitivities and unique responses of the various cells lines, and the nanosilver coating, size, dose, and treatment time that are used.

### 3.1. Fluorescent Dyes Used to Evaluate ROS in Nanosilver Studies

The majority of studies examining the effect of nanosilver on intracellular ROS production use 2′,7′-dichlorodihydrofluorescein diacetate (H_2_DCFDA) [17]. This non-polar dye is thought to enter the cells via diffusion, where it is hydrolysed by esterase enzymes producing 2′,7′-dichlorodihydrofluorescein (H_2_DCF). Intracellular ROS in the form of peroxides oxidizes H_2_DCF, producing 2′,7′-dichlorofluorescein (DCF) which is strongly fluorescent and can be detected. However, there are issues regarding the reliability of this assay, the proposed mechanism, which species of ROS are actually measured, and how much of the result is actually due to artifacts [65,68,70]. Additionally, the cells, media, and the order of treatment used in doing the H_2_DCFDA assay can affect the outcome [77]. A more reliable method is to use a fluorescent dye and flow cytometry with proper gating to remove artifacts due to background fluorescence and cell debris [78]. CellROX fluorescent probes are useful for detecting •O_2_^−^ and •OH. CellROX Green Reagent detects ROS mainly in the nucleus and mitochondria, while CellROX Deep Red and CellROX Orange detects ROS mainly in the cytoplasm. CellROX does not detect H_2_O_2_ well, however, Resorufin is a fluorescent dye specific for H_2_O_2_ [79]. Dihydroethidium (DHE) is another dye used to detect •O_2_^−^ [62], and mitoSOX Red specifically detects mitochondrial •O_2_^−^ [80].

### 3.2. Increase in ROS with Nanosilver Treatment

A significant increase in ROS was seen with both non-toxic and toxic treatment of human embryonic stem cell-derived neural stem/progenitor cells (hESC-derived NPCs) when treated with 13.3 nm citrate-coated nanosilver and measured with the 6-carboxy-2′,7′-dichlorodihydrofluorescein diacetate (carboxy-H_2_DCFDA) dye [3]. Human chronic myeloid leukemia K562 cells exposed to non-toxic treatments of 27 nm PVP-coated nanosilver had a 30–40% increase in intracellular ROS as measured by H_2_DCFDA, increased SOD activity, and a decrease in CAT activity [75]. In NIH 3T3 cells treated with 26.2 nm nanosilver, ROS increased as measured by H_2_DCFDA, intracellular GSH levels decreased, and heme oxygenase-1 (HO-1) gene expression increased [65]. Nanosilver (10 and 20 nm) treatment of TM3 and TM4 cells at the IC_50_ resulted in a significant increase in ROS as measured with H_2_DCFDA, and this increase was somewhat offset by treatment with the antioxidant *N*-acetylcysteine (NAC). A decrease in mitochondrial activity and ATP production was observed alongside an increase in ROS as measured with H_2_DCFDA and DHE in nanosilver (starch-coated, 6–20 nm) treated U251 and IMR-90 cells [62]. One of the downstream effects of the induced oxidative stress that results from the nanosilver treatment was increased cellular damage and membrane leakage as determined with the lactate dehydrogenase (LDH) assay [68]. ROS measured with H_2_DCFDA increased in MCF-7 breast adenocarcinoma cells with non-toxic nanosilver (10–30 nm) treatment [81]. Similarly, ROS measured with carboxy-H_2_DCFDA increased in human renal proximal tubular epithelial (HK-2) cells treated with nanosilver (7.5 nm) treatment conditions resulting in approximately 60% cell viability [82]. Human lung carcinoma (A549) cells treated with 10 nm nanosilver at the EC_50_ increased the amount of ROS measured by H_2_DCFDA fluorescence, however, hypoxia pre-treatment reduced the amount of oxygen present and attenuated this increase in ROS [83]. Nanosilver (18 nm) treatment at the EC_25_ increased the levels of ROS in human ovarian cancer (A2780) cells as measured with H_2_DCFDA. Cytotoxicity, indicated by LDH release, also increased with this treatment. The level of MDA increased, and the levels of GSH, SOD, and CAT significantly decreased in cells treated with nanosilver at the EC_25_ concentration, indicating the induction of oxidative stress [72]. GSH depletion was also observed in HepG2 cells treated with 10 and 75 nm, citrate and PVP-coated nanosilver. The 10 nm citrate coated nanosilver resulted in the highest GSH depletion, followed by the 10 nm PVP-coated nanosilver, 75 nm citrate-coated nanosilver, and finally 75 nm PVP-coated nanosilver [50]. In N27 neurons, the amount of GSH increased with increasing nanosilver treatment with 10 and 75 nm PVP-coated nanosilver. Additionally, as another test for oxidative stress, the level of nitrous oxide was examined through measuring the levels of its metabolite, nitrite, in the cells; and increased nitrite levels were found [69]. ROS increased in K562 cells with non-toxic nanosilver (27 nm, PVP-coated) treatment as measured with H_2_DCFDA; and the activity of SOD increased while the activity of CAT decreased [75]. Manshian et al. [17] measured ROS using CellROX green, and found only a slight increase in ROS due to toxic nanosilver treatment for both C17.2 cells and HUVEC cells treated with 4.2 nm mercaptoundecanoic acid (MUA) or dodecylamine-modified poly(isobutylene-*alt*-maleic anhydride (PMA) coated nanosilver; with only the PMA-coated nanosilver resulting in a significant increase in ROS at the highest treatment concentration [17].

In vivo, the effect of prolonged nanosilver (PVP-coated, 20–30 nm) exposure was examined in male Sprague Dawley rats by Blanco et al. [74]. The mice were treated with 0, 50, 100, and 200 mg/kg/day doses every day for 90 days by gavage administration, and the effects on their livers were assessed. The activities of SOD and CAT increased with dosage until the highest dose, where it decreased. Lipid peroxidation also increased, together indicating a hepatic response to increased ROS [74].

### 3.3. No Change or a Decrease in ROS with Nanosilver Treatment

In contrast to the above findings, no change, or a decrease in ROS production, due to nanosilver exposure is observed in several other studies. No increase in ROS was observed using the H_2_DCFDA dye in HepG2 and human colon cancer (Caco-2) cells, even at nanosilver (20 nm; citrate-coated) concentrations that caused significant cytotoxicity, DNA damage, and mitochondrial injury to the cells [84]. With non-toxic nanosilver treatment, MEL cells exposed to 25 nm PVP-coated nanosilver did not lead to any increase in ROS production as measured with H_2_DCFDA [13,22]. Similarly, in HEK 293T cells treated with non-toxic levels of PVP-coated 25 nm nanosilver, there was no significant increase in ROS as measured with H_2_DCFDA [14]. Human gingival fibroblasts (HGFs) treated with 30 nm nanosilver (in a solution of Chitlac) decreased the production of ROS compared to the untreated cells as measured with chloromethyl-H_2_DCFDA (CM-H_2_DCFDA) [85]. As well, there was no significant increase in ROS as measured using CM-H_2_DCFDA in human neutrophils isolated from blood samples from healthy donors and treated with non-toxic levels of nanosilver (20 and 68.5 nm; citrate-coated) [86]. ToxTracker mouse embryonic stem cell lines containing reporters for various pathways and treated with non-toxic nanosilver (10 and 40 nm; citrate-coated) treatment showed no increase in ROS production as measured with H_2_DCFDA [20]. In liver mitochondria isolated from male Wistar rats treated with nanosilver (<100 nm, 100 µg/kg/day) by gavage administration for 21 days, no increase in ROS was observed in the mitochondria using H_2_DCFDA, and nanosilver treatment did not significantly decrease the ratio of GSH to its oxidized form, glutathione disulfide (GSSG) [87].

Thus, the majority of the studies indicate an increase in ROS and oxidative stress as a result of nanosilver treatment. However, this has been challenged in some studies where no increase in ROS was seen [13,14,20,22,84,85,86,87]. This may be due to different experimental conditions or due to problems detecting the actual levels ROS with H_2_DCFDA, the main dye that is used to detect ROS.

## 4. Nanosilver and Inflammation

Inflammation is an immune response to stress or injury in which leucocyte cells infiltrate the damaged tissue and mount an immune defence and aid in healing. Acute and short term inflammation is beneficial; however, chronic inflammation may lead to damage and diseases such as arthritis and cancer [88,89]. Cytokines such as interleukin-6 (IL-6), IL-1β, tumour necrosis factor-α (TNF-α), interferon-γ (IFN-γ), and transforming growth factor-β (TGF-β) are produced by the leucocyte cells and stimulate changes in the production of acute phase plasma proteins as well as many other biochemical and physiological changes. In acute inflammation, leucocytes are primarily neutrophils, while in chronic inflammation macrophages and lymphocytes are recruited approximately 48–96 h after initiation to aid in destroying the inflammatory agent and promote healing [88,90]. The nuclear factor kappa B (NF-κB) pathway is involved in the cellular response to various stresses including oxidative stress and is involved in the start of inflammation. Phosphorylation of I-κB kinases (IKK), in response to ROS, leads to the release of NF-κB dimers from inhibitory I-κB proteins in the cytosol, allowing them to enter the nucleus and activate gene expression leading to an inflammatory cellular response [91]. Cyclooxygenase-2 (COX-2) is a pro-inflammatory mediator during acute inflammation and is induced by cytokines such as IL-1β and TNF-α [90,92]. The activator protein 1 (AP1) transcription factor family consists of 18 dimers made from Fos, Jun, Maf, or activating transcription factor (ATF) family proteins. Phosphorylation of AP1 regulates its activity. AP1 is activated in response to various stimulants such as inflammatory cytokines, cellular stress, infection, or UV radiation; and once activated, AP1 has a role in various cellular responses including inflammation, cell survival, differentiation, proliferation, and apoptosis [93].

### 4.1. Nanosilver and Inflammation In Vitro

The NF-κB and AP1 pathways were activated by nanosilver (10 and 75 nm, citrate and PVP-coated) in stable luciferase-reporter HepG2 cells [50] and in N27 neurons [69]. Non-toxic nanosilver (citrate-coated, 5 nm) treatment activated the NF-κB pathway in human cervical carcinoma cells (HeLa) and A549 cells; and an increase in the cellular immune response was seen as an increase in the pro-inflammatory cytokine IL-1α [91]. The levels of IL-6 and IL-8 increased in hMSCs treated with non-toxic nanosilver (46 nm) [57]. A slight increase in prostaglandin E2 and a significant increase in IL-6 was observed in HGFs treated with 30 nm nanosilver (in a solution of Chitlac) [85]. Additionally, non-toxic nanosilver treatment (PVP-coated; 10, 50 and 100 nm) resulted in activation of the Nod-like receptor protein 3 (NLRP3)-inflammasome via activated caspase-1 and increased IL-1β secretion in HepG2 cells, with the 10 nm nanosilver being the most potent [63].

### 4.2. Nanosilver and Inflammation In Vivo

Nanosilver treatment has been seen to activate the immune system and cause inflammation in mice. In a study by Park et al. [94], male and female Institute of Cancer Research (ICR) mice orally treated with nanosilver (22, 42, 71 nm) at 1 mg/kg for 14 days resulted in an increase in TGF-β in the serum and increased distribution of B cells and natural killer cells, although the body weight of the mice did not change. Larger (323 nm) silver particles did not cause any significant effect. Longer treatment (28 days) of the mice with 42 nm nanosilver at various doses (0.25, 0.5 and 1 mg/kg) only showed adverse effects in the liver and kidney at the highest dose (specifically, increased levels of alkaline phosphatase, aspartate transaminase, and alanine transaminase; with the latter only being observed in the female mice). An increase in the levels of TGF-β, IL-1, IL-4, IL-6, IL-10, IL-12, and immunoglobulin E (IgE) antibody was observed in the plasma at the higher treatment doses, and the B cell distribution also increased [94]. Nanosilver injected around tumors (made from murine lung squamous tumor cells [KLN 205] injected into female immune competent DBA/2 mice and immune deficient NOD SCIDγ mice) resulted in inflammation in the immune competent DBA/2 mice as visualized with an inflammation-activatable probe (Cat B 750 FAST) and 3D optical imaging [91]. Inflammation has also been observed in the liver tissue of male Sprague Dawley rats treated with PVP-coated nanosilver (22.32 nm) by intraperitoneal injection [67].

### 4.3. Anti-inflammatory Properties of Nanosilver in Wound Healing

The process of skin wound healing involves inflammation, proliferation, and tissue remodelling. The injury stimulates inflammation and the release of pro-inflammatory cytokines. In proliferation, granulation tissue formation and angiogenesis occur, and are aided by the macrophages. During tissue remodelling, damaged tissue is removed and the extracellular matrix is remodelled, with this final process being controlled by various matrix metalloproteinases (MMPs) and tissue inhibitors [90]. Nanosilver treatment has been found to be beneficial in wound healing [95,96,97,98,99] since the induced short term inflammation quickens the healing process [90,100]. In a skin wound healing model with normal human dermal fibroblasts (NHDFs) and normal human epidermal keratinocytes (NHEKs), nanosilver (10 nm) treatment decreased the expression of TNF-α, IL-12, COX-2, vascular endothelial growth factor (VEGF), and MMP-3, thus serving to speed up the healing process [90]. In a thermal injury animal model using male BALB/C mice, bandages coated with nanosilver (14 nm) decreased the inflammation, eliminated bacterial growth, and resulted in faster healing with reduced scarring compared to the control mice. Nanosilver treatment also affected the mRNA expression of various cytokines: IL-6 was downregulated, while IL-10, VEGF, and IFN-γ were all upregulated. TGF-β1 was initially upregulated, before being downregulated later in the healing process [95]. One millilitre of various concentrations (9, 45, and 90 µM) of 9.3 nm nanosilver were put in a wound in BALB/C mice before being surgically closed, and it was found that the highest concentration of nanosilver greatly decreased the severity of postoperative peritoneal adhesions and inflammation. In cell culture studies using mouse macrophage cell lines (RAW264.7 and J774.1), nanosilver treatment was able to decrease the level of TNF-α produced as a result of lipopolysaccharide (LPS) induced inflammation [96]. Studies have been conducted combining nanosilver treatment with other compounds and natural extracts, and these have resulted in increased speed in wound healing [97,100,101,102]. Since inflammation is an important step in the wound healing process, the initial short-term increase in inflammation due to the nanosilver treatment reported in the in vitro and in vivo studies leads to an increased speed of wound healing and a faster decrease in inflammation, which agrees with the results found in the wound healing studies.

## 5. Nanosilver and Hypoxia Stress

Low oxygen (hypoxic) conditions are encountered by humans during various physiological (high altitude), developmental (during embryogenesis), and clinical conditions (during embryogenesis, cardiac arrest, stroke, and in solid tumors) [103]. The cellular response to hypoxia involves the activation of the transcription factor hypoxia-inducible factor (HIF). Under normal oxygen (normoxic) conditions, the alpha subunit of HIF (HIF-α) is hydroxylated by oxygen-dependent prolyl hydroxylase enzymes, leading to the recognition of HIF-α by E3 ubiquitin ligases, and its subsequent ubiquitination and proteasomal degradation. For complete HIF-α activation, the HIF-α terminal transactivation domain must be hydroxylated by Factor Inhibiting HIF (FIH), an asparaginyl/aspartic acid hydroxylase, which blocks the coactivator CREB-binding protein/p300 from binding to HIF-α. Under hypoxic conditions, HIF-α hydroxylation and degradation is inhibited, and HIF-α translocates to the nucleus where it binds to HIF-β (also known as the aryl hydrocarbon receptor nuclear translocator (ARNT)). This heterodimer binds to the hypoxic response elements (HRE) in the promoters of HIF target genes, thus activating gene expression integral for adaptation to hypoxic stress [60,103,104].

Only a few studies have focused on the effects of nanosilver treatment on HIF-1α expression, and on the effects of nanosilver treatment in combination with hypoxia treatment. In A549 cells, HIF-1α protein expression was found to increase in hypoxic conditions and in EC_50_ level nanosilver treatment [83]. Similarly, an increase in HIF-1α expression was observed in hMSCs with non-toxic nanosilver treatment [105]. Gene expression studies with C17.2 cells treated with non-toxic nanosilver concentrations resulted in the upregulation of several HIF target genes: adrenomedullin, HO-1, and serpine1 [17]. The levels of VEGF increased with non-toxic nanosilver (46 nm) treatment on hMSCs [57]. As well, two studies on nematodes (*Caenorhabditis elegans*) also reported an increase in HIF-1α activation with nanosilver exposure [106,107]. Yang et al. [60] observed decreased protein expression of HIF-1α, vascular endothelial growth factor-A (VEGF-A), and glucose transporter type 1 (GLUT1) in human breast cancer MCF-7 cells that were treated with a combination of hypoxia and non-toxic nanosilver treatment, as compared to cells treated only with hypoxia [60]. In an in vivo study using female BALBc mice treated with ovalbumin inhalation to model allergic airway inflammation, nanosilver treatment reduced the effects of the ovalbumin treatment by lowering the expression of HIF-1α and VEGF [108]. More research needs to be done to understand the effects that nanosilver treatment has on the hypoxic response pathway and on HIF-1α expression.

## 6. Nanosilver and the Mitochondria

Mitochondria are the cellular site of ATP production through oxidative phosphorylation [109]. The mitochondria forms a complex reticular network throughout the cytosol, allowing communication between it and other organelles [110]. Mitochondrial mediated intrinsic apoptosis involves active p53 instigating the release of cytochrome C from the mitochondria, followed by a caspase signalling cascade involving caspase-9. Extrinsic death receptor apoptosis involves death receptors binding to caspase-8. Caspase-8 and -9 are initiator caspases that activate the executioner caspases, caspase-3 and -7 [68,110,111]. Inhibition of caspase-8 and -9 decreases apoptosis resulting from nanosilver (14 nm; PVP-coated) treatment of rat pheochromocytoma (PC12) cells, indicating that nanosilver triggers both the mitochondrial and the extrinsic apoptotic pathways [112].

A study that examined primary rat cerebellar granule cells (CGC) treated with various combinations of *N*-methyl-d-aspartate (NMDA) receptor agonists, NMDA receptor antagonists, and nanosilver, found that nanosilver treatment increased intracellular calcium levels through NMDA receptor activation [113]. This is of note since this influx of calcium into the neuron stimulates calcium induced calcium release (CICR), as well as inositol 1,4,5-triphosphate receptors (IP3R)-mediated calcium release from the endoplasmic reticulum (ER) calcium reserves into the cytoplasm, leading to increased mitochondrial calcium levels and potentially to mitochondrial dysfunction [113,114]. Nanosilver treatment of human Chang liver cells at the EC_50_ increased the mitochondrial calcium level in the cells by over two-fold, as was seen by flow cytometry with the Rhod2-AM fluorescent probe [115]. Rhod2-AM sequesters preferentially into the mitochondria due to its positive charge, as well as only fluorescing once it is oxidized which generally occurs in the mitochondria [115], thus making it useful in detecting mitochondrial calcium levels.

Mitochondria normally have a negative electric potential across the inner mitochondrial membrane, and disruption of mitochondrial homeostasis leads to depolarization and decrease in the membrane potential [84]. Mitochondrial membrane potential depolarization was seen via 5,5′,6,6′-Tetrachloro-1,1′,3,3′-tetraethylbenzimidazolocarbocyanine iodide (JC-1) mitochondrial staining once the nanosilver (31.1 nm; PVP-coated) treatment reached the EC_50_ level for the SH-SY5Y cells. Along with this, decreased ATP production was seen [116]. The BCL2 family of proteins is involved in mitochondrial mediated apoptosis; with BAX and BAK being pro-apoptotic proteins, and BCL2 being anti-apoptotic [72]. With nanosilver (31.1 nm; PVP-coated) treatment on SH-SY5Y cells, increased BAX/BCL2 protein ratio, and increased protein levels of the mitochondrial mediated apoptotic proteins: caspase-3, cleaved caspase-3, cytochrome c, and cleaved caspase-9 were observed [116].

In A549 cells, the mitochondrial membrane potential as seen with JC-1 staining decreased when the cells were treated with 10 nm nanosilver at the EC_50_ level. As well, nanosilver treatment increased mitochondrial mediated apoptosis through an increase in activated caspase-3. Pre-exposure with hypoxia lessened the effects of the nanosilver treatment on the mitochondrial membrane potential and the mitochondrial damage [83]. A decrease in mitochondrial membrane potential was also seen with JC-1 staining in A2780 treated with nanosilver (18 nm) at the EC_25_ [72]. Mitochondrial mediated apoptosis was induced as seen by an increase in BAX and BAK mRNA, a decrease in BCL2 mRNA, an increase in caspase-9 and caspase-3 mRNA, and a decrease in pro-caspase-3 mRNA expression. Furthermore, the use of a caspase-3 inhibitor successfully blocked the increased caspase-3 activity due to the nanosilver treatment [72]. Similarly, under high nanosilver treatment, caspase-7 and caspase-9 had increased processing and activity in MCF-7 cells [117]. However, nontoxic nanosilver treatment of human bronchial epithelial (16-HBE) cells did not lead to mitochondrial mediated apoptosis since cleaved caspase-3 was not detected in these cells [118]. Reduced mitochondrial membrane potential was indicated by a decrease in retention of the positively charged rhodamine 123 fluorescent dye in HepG2, Caco-2 [84], and CGC cells [113,119]. The HepG2 cells were more sensitive and responded to lower dose nanosilver treatment, whereas the Caco-2 and CGC cells only showed a decrease in membrane potential at a dose that corresponded to the EC_50_ of the cells [84,113,119]. Another fluorescent cationic dye, MitoTracker Red, also showed a decrease in mitochondrial membrane potential with high nanosilver treatment in human colon carcinoma cells (HCT116) [120]. Mitochondria mediated apoptosis was triggered by high dose nanosilver treatment, as indicated by increased levels of activate phosphorylated c-Jun N-terminal Kinase (JNK), the translocation of the pro-apoptotic protein BAX to the mitochondria, and cytochrome C release into the cytoplasm [120,121]. Visible mitochondrial damage was caused in TM3 and TM4 cells treated with 10 and 20 nm nanosilver at the EC_50_, and mitochondrial mediated apoptosis was induced as seen with an increase in caspase-3, caspase-8, and caspase-9 mRNA expression [68].

In in vivo studies, impaired energy metabolism was seen as a drastic decrease in ATP production in liver tissue from male Sprague Dawley rats injected intraperitoneally with nanosilver (PVP-coated, 22.32 nm). Apoptosis increased with the nanosilver treatment (as seen by an increase in cleaved caspase-3), and DNA fragmentation also was found (as seen with the terminal deoxynucleotidyl transferase dUTP nick end labeling (TUNEL) assay) [67]. Liver mitochondria from male Wistar rats treated with nanosilver (<100 nm, 100 µg/kg/day) by gavage administration for 21 days resulted in mitochondrial swelling and a decrease in the ADP consumption (as measured by oxygen electrode), indicating a decrease in oxidative phosphorylation [87].

Thus, high dose nanosilver treatment around the EC_50_ value for the cells leads to increased mitochondrial dysfunction, mitochondrial-mediated apoptosis, and decreased ATP production.

## 7. Nanosilver and Endoplasmic Reticulum Stress (Unfolded Protein Response)

The ER is an organelle that forms a reticular network throughout the cytosol, and is the main site in the cell for protein synthesis and folding, as well as being vital for lipid biogenesis and calcium storage [116,122]. It is in close communication with the mitochondria for the purpose of the transfer of ions, proteins, and lipids. Various cellular stresses such as hypoxia, oxidative stress, low glucose levels, viral infection, pharmaceuticals, or environmental stressors can cause unfolded or misfolded proteins to build up in the ER, a situation that is described as ER stress [122,123,124]. ER stress will activate the unfolded protein response (UPR) signalling pathway to return the cell to its healthy state, or if this is not possible, to lead to cell death. Pro-survival mechanisms include reducing general protein synthesis, increasing the number of chaperones produced in order to alleviate the ER stress, and increasing protein degradation [125,126]. Protein kinase RNA-like ER kinase (PERK), ATF-6, and inositol-requiring enzyme-1α (IRE-1α) are three ER membrane spanning proteins that act as sensors for ER stress. They are inactive when bound to GRP78, an ER luminal chaperone [117,123]. Under ER stress, GRP78 dissociates from these complexes to assist in protein folding, thus activating the three ER sensors and their respective UPR pathways [123].

Release of PERK from GRP78 and HSP90 chaperones allows PERK to become active through homodimerization and autophosphorylation, allowing PERK to phosphorylate eukaryotic translation initiation factor 2 (eIF2-α) and inhibit protein translation [123,124]. If the ER stress persists, the PERK/eIF2-α pathway can trigger apoptosis by increasing the translation of mRNAs for certain transcription factors such as ATF-4. ATF-4 regulates many genes integral to cell survival and the cellular stress response, but under these conditions of prolonged ER stress, ATF-4 stimulates the translation of CCAAT/enhancer-binding protein-homologous protein (CHOP or GADD153), which initiates apoptosis and suppresses the transcription of anti-apoptotic BCL2 family proteins [115,117,127].

Release of ATF-6 from GRP78 allows this transcription factor to be transported to the Golgi apparatus and activated by proteolytic cleavage by transmembrane Site-1 and Site-2 proteases, allowing the N-terminal fragment of ATF-6 to then stimulate the needed gene expression either for survival (such as ATF-4 and X-box binding protein 1, XBP1) or for cell death [117,124,126].

Release of IRE-1α from Grp78 and HSP90 allows this kinase/endoribonuclease enzyme to become active through homodimerization and autophosphorylation. Active IRE-1α excises an intron from XBP1 mRNA allowing it to be translated into XBP1 protein, a transcription factor that induces the expression of various target genes such as Grp78 [115,123]. IRE-1α can also phosphorylate and activate JNK. Active JNK phosphorylates BCL2 family proteins suppressing apoptosis [117].

Recently within the last six years, novel research has been published on the effects of nanosilver on ER stress and the UPR. Treatment of human Chang liver cells with nanosilver (≤100nm) at a high concentration corresponding to the EC_50_ value of the cells initiated the UPR with increased protein levels of released chaperone GRP78, phosphorylated PERK, phosphorylated eIF2-α, phosphorylated IRE-1, spliced XBP1, cleaved ATF-6, CHOP, and caspase 12 [115]. Additionally, siRNA knockdown of PERK, IRE-1α, ATF-6, and CHOP reduced the intensity of the ER stress resulting from nanosilver treatment [115]. Similar results were seen in SH-SY5Y cells treated with nanosilver (PVP-coated, 31.1 nm) up to the EC_50_ value of the cells, in addition to an increased BAX/BCL2 ratio and increased cleaved caspase 3 for the apoptotic regulator proteins [116]. Increased mRNA levels were also seen for GRP78, XBP-1, and CHOP; further evidence that high nanosilver treatment induces ER stress and the UPR [116]. Treatment of MCF-7 and T-47D breast cancer cells with high nanosilver (2 and 15 nm) concentrations increased the protein levels of phosphorylated PERK, phosphorylated eIF2-α, and phosphorylated IRE1α. Inhibitors of ER stress were able to attenuate the toxicity due to the high nanosilver treatments, indicating that the ER stress response pathways were contributing to the cell death [117].

At lower non-toxic nanosilver (20 nm) treatment concentrations, perturbations in ER homeostasis are observed but not cytotoxicity [118]. PERK and HSP70 protein levels were increased with non-toxic nanosilver treatment in THP-1 cells, while no difference in the protein levels of GRPp78 and ATF-6 were observed. Interestingly, ATF-6 degradation and cell death in the THP-1 cells were seen only with very high nanosilver (15 nm) treatments [124]. Non-toxic nanosilver (20 nm, polyoxyethylene glycerol trioleate/Tween 20 stabilized) treatment increased the protein level of GRP78 in 16HBE cells, but not in HepG2 or HUVECs, indicating that 16HBE cells are more sensitive to nanosilver induced ER stress response [118]. Additionally, the 16HBE cells expressed more caspase-12 than caspase-3, and showed cleaved caspase-12; indicating ER mediated apoptosis, while the protein level of JNK increased in all three of the cell lines. The mRNA levels of spliced XBP-1 and CHOP were upregulated in the 16HBE cells, while the HepG2 and HUVEC cells were less responsive with only CHOP mRNA being upregulated in the HepG2 cells and no change being seen in the HUVEC cells [118]. In another study, fluorescence imaging showed the induction of CHOP in HepG2 cells with non-toxic nanosilver (PVP-coated; 10, 50 and 100 nm) treatments [63].

A human UPR PCR array test with 16HBE cells indicated nine genes that were upregulated by more than threefold by nontoxic nanosilver (20 nm, polyoxyethylene glycerol trioleate/Tween 20 stabilized) treatment. These included genes for heat shock proteins: heat shock 70 kDa protein 1 beta (*HSPA1β*), heat shock 105 kDa/110 kDa protein 1 (*HSPH1*), DNAJ (*HSP40*) homolog subfamily B member 9 (*DNAJB9*), and ER stress markers: CHOP, protein phosphatase 1 regulatory (inhibitor) subunit 15A (*PPP1R15A*), homocysteine inducible ER protein with ubiquitin-like domain 1 (*HERPUD1*), adrenomedullin 2 (*ADM2*), asparagine synthetase (*ASNS*), and pseudokinase tribbles homolog 3 (*TRIB3*) [118]. The large number of chaperone proteins upregulated by nontoxic nanosilver treatment is further evidence that these doses initiate the unfolded protein response.

### ER and Mitochondrial Interactions with Nanosilver

The length of membrane in close contact between the ER and the mitochondria was seen to visibly increase with nanosilver (PVP-coated, 31.1 nm) treatment of human neuroblastoma (SH-SY5Y) cells in TEM images. Additionally, ER- and mitochondrial-specific staining showed that the co-localization of the ER and mitochondria increased with nanosilver treatment [116]. The areas where the mitochondria and ER membranes associate are known as mitochondria-associated membranes (MAMs) and these areas are involved in interorganelle communication through various receptors and channels [111]. ER stress is known to cause the release of calcium from the ER to the cytoplasm through IP3R, and this released calcium can further stimulate CICR through the ryanodine receptors in the ER membrane [116,128], which leads to the uptake of calcium into the mitochondria. Additionally, the MAM protein, phosphatase, and tensin homolog deleted on chromosome 10 (PTEN), is thought to increase the release of calcium from the ER by decreasing the phosphorylation of IP3R, and thus increasing the transfer of calcium to the mitochondria [116]. With nanosilver (PVP-coated, 31.1 nm) treatment of SH-SY5Y cells, PTEN moved from the cytoplasm to the ER and MAMs, and co-immunoprecipitation of PTEN with IP3R indicated direct interaction between PTEN and IP3R, as well as showing a decrease in phosphorylated IP3R with toxic nanosilver treatment [116].

## 8. Effects of Nanosilver on Autophagy

Autophagy refers to the cellular-mediated degradation of proteins, sugars, lipids, and some organelles via the lysosome [129]. This degradation may be activated either to remove damaged cellular components, or as a protective response to stress such as starvation, stroke, hypoxia, radiation, or chemotherapy [18,83]. Many different nanoparticles have been found to induce autophagy [18,45]. Autophagy recycles cytosolic components to aid in cell survival, however, prolonged autophagy results in excessive degradation and leads to cell death [18]. There are three different autophagic pathways—macroautophagy, microautophagy, and chaperone-mediated autophagy—with the term ‘autophagy’ generally being used to refer to macroautophagy.

In macroautophagy, the ER is stimulated to form an omegasome, which matures into an isolation membrane (also called a preautophagosome or phagophore), and subsequently elongates to form a structure with a double-membrane called an autophagosome which closes around the components to be degraded. The autophagosome fuses with lysosomes forming an autolysosome, and the contents as well as the inner autophagosome membrane are degraded by lysosomal hydrolases. Permease enzymes in the lysosome membrane release the degradation products back into the cytosol for further cellular use [129,130].

Microautophagy involves the lysosome directly engulfing the components to be degraded without the formation of an autophagosome [129,130].

In chaperon-mediated autophagy, misfolded or unwanted proteins containing the KFERQ motif are recognized by the heat shock cognate 71 kDa protein (HSC70) chaperone, brought into the lysosome by a lysosomal membrane protein, lysosome-associated protein-2A (LAMP2A), and the proteins are subsequently degraded [129,130].

Transcription factor EB (TFEB) is integral to regulating autophagy and lysosomal related genes, such as microtubule associated protein 1 light chain 3 β (*MAPLC3B*), sequestosome 1 (*p62/SQSTM1*), UV radiation resistance-associated gene (UVRAG), WD repeat domain phosphoinositide-interacting protein 1 (*WIPI1*), vacuolar protein sorting-associated protein 11 homolog (*VPS11*), vacuolar protein sorting-associated protein 18 homolog (VPS18), autophagy related 9B (*ATG9B*), and genes in the coordinated lysosomal expression and regulation (CLEAR) network respectively [47,131]. Microtubule-associated protein 1 light chain 3 (LC3) is associated with autophagosome formation, exists as either LC3-I or LC3-II, and is used as a marker for autophagy. LC3-I is a soluble protein found in the cytosol. Under starvation or other autophagy promoting conditions LC3-I is cleaved by autophagin and conjugated to phosphatidylethanolamine, converting it to LC3-II, which is then incorporated into the isolation membrane and autophagosome membrane. Generally, an increase in LC3-II indicates an increase in autophagosome accumulation; however, it must be remembered that LC3-II is degraded with the autolysosomes during normal turnover, and immunoblotting problems may occur [19,132,133]. Both new autophagosome formation and the blockage of autophagosome degradation results in an increase in autophagosomes and LC3-II in the cells, and care must be taken in order to determine whether autophagy induction or the blockage of autophagic flux has indeed occurred [18,134]. In order to determine this, the degradation of p62 can be examined. When p62 binds to LC3, it is incorporated into autophagosomes, and is degraded during autophagy. However, change in p62 protein expression is not specific to autophagy and should not be used exclusively to show changes in autophagy [132]. Inhibition of autolysosomal degradation and turnover, such as with bafilomycin A1 or chloroquine (both of which inhibit the fusion between autophagosomes and lysosomes [4,131]) can also be used to determine if autophagy is truly induced by the treatment of interest, since if this is the case then the level of LC3-II should further increase [19,135].

### 8.1. Studies where Nanosilver Induces Autophagy

Nanosilver (PVP-coated, 26.5 nm) was used to treat HeLa cells that had been stably transfected with LC3 tagged with enhanced green fluorescent protein (HeLa EGFP-LC3) [18]. Control HeLa EGFP-LC3 cells showed a green smear which formed into distinct bright green dots upon non-toxic nanosilver treatment, indicating LC3 aggregation. As well, the green dots for EGFP-LC3 co-localized with acidic vesicles and lysosomes as seen with the monodansylcadaverine and LysoTracker Red dyes respectively. The protein expression of LC3-II increased, TEM imaging (an important method for visualizing autophagosomes [44]) indicated the increased presence of autophagosomes, p62 protein expression decreased, and the use of autolysosomal degradation inhibitors resulted in an increase in LC3-II in nanosilver treated HeLa cells. Together, all these factors indicated that non-toxic nanosilver exposure induces an increase in autophagy. Inhibition of class III phosphatidylinositol 3-kinase (PtdIns3K), a kinase involved in autophagosome formation, by wortmannin, inhibited the nanosilver-induced increase in LC3-II expression; indicating that nanosilver induces autophagy through the PtdIns3K pathway [18].

Active mammalian target of rapamycin complex 1 (mTORC1) phosphorylates TFEB, causing it to be inactive and located in the cytosol. Various stress conditions such as starvation or lysosomal stress will deactivate mTORC1, allowing TFEB to be dephosphorylated and to translocate to the nucleus and regulate gene expression. As well, extracellular signal-regulated kinases 1/2 (ERK1/2) and protein kinase C β (PKC β) are also involved in the phosphorylation of TFEB [47]. Nanosilver treatment did not change the phosphorylation level of mTOR or of its substrate ribosomal protein S6 kinase 70 kDa (P70S6K) indicating that this pathway was not involved. However, Zhu et al. [19] did observe a decrease in phosphorylated mTOR with nanosilver treatment. In order to understand a possible mechanism behind nanosilver-induced cytoprotective autophagy, Lin et al. [47] examined the effects of nanosilver treatment on TFEB. HeLa cells stably transfected with TFEB tagged to enhanced green fluorescent protein (EGFP-TFEB) were treated with nanosilver (PVP-coated, 26.8 nm) at non-toxic levels, and TFEB was seen to locate in the nucleus. The mRNA expression of the autophagy related genes MAP1LC3B and p62 were also upregulated. Treatment with the mTOR inhibitor, Torin 1, also resulted in the translocation of TFEB to the nucleus. However, nanosilver did not appear to inactivate mTORC1, ERK1/2, or PKC in this study. A time course experiment indicated that nanosilver-induced TFEB translocation preceded an increase in LC3-II protein expression, and knockdown of TFEB decreased the increase in LC3-II. As well, inhibition of autophagy by 3-methyladenine (3-MA) did not affect the nanosilver induced translocation of TFEB to the nucleus. Nanosilver-induced apoptosis increased in the HeLa cells with both autophagy inhibition by 3-MA and TFEB knockdown, indicating the mechanism by which nanosilver induced autophagy was cytoprotective. Co-treatment of the cells with both nanosilver and the antioxidant NAC attenuated TFEB translocation, demonstrating the importance of ROS in the nanosilver-induced autophagy in HeLa cells [47]. Similarly, in human U251 glioblastoma cells, treatment with PVP-coated nanosilver (2.43, 15.47, 40.05 nm) induced autophagy in the cells [45]. This was indicated by an increase in autophagosomes identified in TEM images, an increase in florescent dots when the cells were stained with an autophagosome dye (Cyto-ID Green), the co-localization of many of the Cyto-ID fluorescent dots with that of Lyso Tracker Red indicating the presence of autolysosomes, an increase in LC3-II protein expression, and the degradation of p62. The induction of autophagy in response to nanosilver treatment was a protective mechanism for the cells, since co-treatments of nanosilver with 3-methylamphetamine (3-MA) inhibited autophagy by inhibiting PtdIns3K and resulted in increased cell death. Additionally, the ERK and JNK pathways were found to be involved in the process of nanosilver-induced autophagy since inhibitors for JNK and ERK inhibited autophagy [45]. Acidic vesicular organelles (AVOs) can be detected via acridine orange (AO) dye which accumulates in acidic vesicles [65,136,137]. Autophagy was increased in U251 glioblastoma cells with non-toxic nanosilver (citrate-coated, 15.26 nm) treatment as seen with an increase in AVOs and LC3. Nanosilver and radiation treatment combined further increased autophagy in the cells as a survival mechanism in response to the treatments [66]. An increase in the number of AVOs were also observed in MCF-7 cells treated with non-toxic levels of 10–30 nm nanosilver [81]. Further studies with U251 glioblastoma cells by Wu et al. [46] indicated that non-toxic nanosilver (PVP-coated, 15.38 nm) treatment produced an increase in ROS as measured with H_2_DCFDA, and this increase in ROS triggered autophagy, decreased mitochondrial membrane potential, and increased apoptosis due to radiation exposure. Antioxidant treatment with NAC or vitamin C attenuated each of these effects: decreasing the amount of ROS, LC3-II protein expression, the number of autophagosomes, and decreasing the apoptosis caused by nanosilver and radiation treatment. The inhibition of autophagy with 3-MA decreased this cell survival mechanism and lead to increased ROS and caspase-3 activity, which increased the cell death of the glioma cells due to nanosilver and radiation [46].

Ba/F3 treated with toxic levels of PVP-coated, 11.17 nm nanosilver just above the EC_50_ level, induced autophagy in a ROS dependent manor [19]. An increased number of autophagosomes were seen with TEM, and the protein expression of LC3-II increased. Interestingly, the increase in LC3-II appeared to be nanosilver-specific since this effect was not induced by silver ions. Inhibition of autolysosome degradation with chloroquine further increased the level of LC3-II, and the protein expression of p62 decreased with nanosilver treatment, indicating that autophagy was induced. As an additional check for normal autophagic flux, the activity of a lysosome enzyme, acid phosphatase, was examined and found to be not affected by nanosilver treatment, indicating that normal lysosomal degradation occurred. ROS increased with nanosilver treatment as seen with the H_2_DCF-DA dye, and pre-treatment with the antioxidants NAC or vitamin C decreased autophagy indicating that oxidative stress was upstream of this process. Nanosilver treatment greatly decreased the protein expression of active phosphorylated mTOR, and antioxidant pre-treatment relieved this inhibition [19].

Many of the *ATG* genes are involved in autophagosome formation [129]. Several of the autophagy proteins are also involved in activating apoptosis, including ATG3, ATG5, ATG6/Beclin1, ATG7, ATG10, ATG12, and ATG17 [72,130]. Inhibition of autophagy by wortmannin or *ATG5* knockdown increased the nanosilver induced cell death in HeLa cells indicating that the process of autophagy was aiding in cell survival [18]. Inhibition of autophagy with *ATG5* knockdown or 3-MA co-treatment reduced the apoptosis due to the nanosilver treatment around the EC_50_ concentration. Thus, in this study, the toxic nanosilver treatment induced ROS, which inactivated mTOR, leading to the induction of autophagy, and cell death in the Ba/F3 cells [19].

Nanosilver aggregates were observed inside autophagosomes, and the protein expression of LC3-II increased in HGFs treated with 30 nm nanosilver (in a solution of Chitlac) [85]. Autophagosome accumulation and increased LC3-II protein expression was seen in HepG2 cells treated with non-toxic nanosilver treatment (PVP-coated, 10, 50 and 100 nm). Additionally, pre-treatment with the autophagosome inhibitor, 3-MA, decreased the expression of LC3-II. Increased nanosilver treatment concentrations resulted in increased lysosome activity and dysfunction, and toxic nanosilver treatment resulted in an increase in caspase-3 activity and apoptosis. As expected, the strength of the effect elicited by the nanosilver decreased as the size of the particle increased [63].

The expression of several the *ATG* genes has been found to change in cells treated with nanosilver. In a study by Zhang et al. [68], TM3 and TM4 cells treated with nanosilver (10 and 20 nm) at the EC_50_ had an increased number of autophagosomes as visualized with TEM. In the TM3 cells the mRNA expression for *ATG7* and *ATG8* were upregulated while *ATG6* was downregulated; and in TM4 cells *ATG6* and *ATG7* were upregulated while *ATG8* was downregulated [68]. Nanosilver (18 nm) treatment of A2780 at the EC25 level induced the formation of many vacuoles and autophagosomes seen with TEM, as well as the upregulating the mRNA expression of *ATG5* and *ATG7*, and downregulating the mRNA expression of *ATG3*, *ATG6*, and *ATG10* [72]. An increase in LC3 fluorescence and the upregulation of the autophagy genes *ATG12* and Beclin1 was observed in C17.2 cells treated with non-toxic nanosilver treatment using 4.2 nm MUA or dodecylamine-modified poly(isobutylene-*alt*-maleic anhydride (PMA) coated nanosilver [17].

In an in vivo experiment, male Sprague Dawley rats exposed to nanosilver (PVP-coated, 22.32 nm) via a one-time interperitoneal injection of 500 mg/kg had visible phagophores, autophagosomes, and autolysosomes in their liver tissue. The expression of LC3-II as well as the presence of LC3-II protein aggregates increased in the liver tissue the day after exposure and then decreased again over time [67]. In another study, the levels of LC3-I and LC3-II both increased with increasing nanosilver dose, but the ratio of LC3-II to LC3-I only increased at the highest treatment of 200 mg/kg/day in male Sprague Dawley rats exposed to PVP-coated nanosilver (20–30 nm) over 90 days by gavage administration [74].

### 8.2. Studies where Nanosilver Blocks Autophagic Flux

In contrast to inducing autophagy, nanosilver treatment resulted in a blockage in autophagic flux under the experimental conditions used in the following studies. NIH 3T3 cells treated with 26.2 nm nanosilver had an increase in the number of autophagosomes as seen with TEM microscopy, an increase in cytosolic AVOs, and an increase in LC3-II protein expression. However, the protein expression of p62 also increased indicating that the nanosilver treatment inhibited autophagosome turnover [65]. THP-1 monocytes also showed a blockage in autophagic flux with nanosilver treatment [134]. Autophagy is essential for the process of monocyte differentiation into macrophages, and differentiation stimulated by phorbol 12-myristate 13-acetate (PMA) was inhibited by the nanosilver (>30 nm) treatment. The protein expression of p62 increased along with the protein expression of LC3-II and the number of autophagosomes with PMA and nanosilver treatment, and degradation of p62 did not occur. Additionally, lysosome membrane stability decreased and lysosomal pH alkalization increased with nanosilver treatment, indicating lysosomal dysfunction in the THP-1 monocytes which may have played a part in the observed blockage in autophagic flux [134]. A549 cells treated with toxic levels of nanosilver (10 nm) at the EC_50_ showed an increase in the number of autophagosomes and autolysosomes, increased mitochondrial damage, and increased ATG5, LC3-II, and p62 protein expression levels; which indicated a blockage in autophagic flux in these cells. Interestingly, pre-exposure of the A549 cells to hypoxia before the EC_50_ nanosilver treatment reduced the nanosilver-induced increase in p62, while still increasing ATG5 and LC3-II [83].

HIF-1α is another gene essential to the autophagy process [83]. HIF-1α knockdown decreased the nanosilver (10 nm) induced increase in the levels of ATG5, LC3-II, and p62 in A549 cells, indicating the importance of HIF-1α in mediating autophagy [83]. In another study, non-toxic nanosilver (citrate-coated, 69.8 nm) treatment of A549 cells increased the number of autophagic vacuoles seen with Cyto-ID fluorescence and increased the protein expression of LC3-II [131]. This increase was due to a blockage in autophagic flux since the level of p62 also increased, and inhibition of autophagy by bafilomycin A1 did not cause a further increase in LC3-II. In contrast to the study by Lin et al. [47], nanosilver treatment decreased the protein and mRNA expression of TFEB, and overexpression or knockdown of TFEB in nanosilver treated cells did not change the level of LC3-II. Lysosomal pH was found to increase indicating that lysosomal dysfunction occurred in this study [131].

Nanosilver-induced blockage of autophagic flux was also seen in human pulmonary mucoepidermoid carcinoma (NCI-H292) cells treated with nanosilver (citrate or lipoic acid coated; 10 nm), where an increased number of autophagosomes were seen, as well as increased protein expression of LC3-II and p62 [4]. An alternative autophagy pathway not involving the conversion of LC3-I to LC3-II may also occur. This pathway involves the formation of autophagosomes from the fusion of the isolation membrane with late endosomal and *trans-*Golgi vesicles regulated by the GTPase Ras-related protein 9 (Rab9) [4,138,139]. Nanosilver (citrate or lipoic acid coated; 10 nm) treatment of transformed human bronchial epithelial (BEAS-2B) cells increased the number of autophagosomes containing Rab9, indicating that alternative autophagy was induced even though conventional autophagy was blocked by the nanosilver treatment [4].

## 9. Nanosilver and Angiogenesis

Angiogenesis is the natural process of new blood vessels growing from the capillary endothelium of established blood vessels [140,141]. This is especially important during physiological growth and development, or during wound healing [142]. An imbalance in the expression of pro-angiogenic growth factors such as VEGF, acidic fibroblast growth factor (FGF-1), basic fibroblast growth factor (FGF-2), and angiopoietin; versus angiogenesis inhibitors such as endostatin and angiostatin, may lead to various ischemic, ocular, and inflammatory diseases [142,143]. The process of angiogenesis involves the production of growth factors, followed by VEGF and FGF-2 stimulated release of proteases and plasminogen activators from the endothelial cells to degrade the basement membrane of the blood vessel, cell migration into the surrounding tissue, and cell proliferation to form the new blood vessels [142,144].

One of the pathways mediated by VEGF in angiogenesis is the phosphatidylinositol 3-kinase (PI3K)/protein kinase B (AKT) signaling pathway. VEGF binds to and activates the VEGF receptor (VEGFR, also known as KDR or Flk-1), a type III receptor tyrosine kinase, which in turn activates phosphatidylinositide 3-kinase (PI3K), which leads to the phosphorylation and activation of AKT, a serine/threonine kinase involved in cell survival and the mTOR pathway [142,144]. Gurunathan et al. [142] found that treatments of bovine retinal endothelial cells (BREC) with nanosilver (40–50 nm) up to the EC_50_ value for the cells decreased angiogenesis via inhibition of the PI3K/AKT pathway. Treatment of the cells with VEGF was used to induce angiogenesis, and co-treatments of nanosilver and VEGF greatly decreased the VEGF induction of angiogenesis. Nanosilver treatment dramatically decreased VEGF induced cell proliferation, cell migration, capillary-like tube formation, PI3K activity, and AKT phosphorylation [142]. In a following study, nanosilver EC_50_ treatment of the BREC cells induced apoptosis through caspase-3 activation [145]. In a study using HUVEC cells, toxic nanosilver treatment with an average size of 10 nm resulted in greatly decreased protein levels of VEGF in the cells, as well as endothelial tube formation [60].

Sheikpranbabu et al. [146] examined the effects of non-toxic 50 nm nanosilver treatments on endothelial cell permeability using porcine retinal endothelial cells (PREC). VEGF stimulates endothelial cell permeability, as does the inflammatory cytokine IL-1β. Src kinase is also involved in angiogenesis, and higher levels of active phosphorylated Src kinase are found with VEGF induced vascular permeability [147]. Non-toxic nanosilver treatment significantly inhibited VEGF and IL-1β from increasing the levels of phosphorylated Src kinase, and in this way decreased the VEGF and IL-1β induced permeability of the PREC cells [146]. In terms of endothelial cell viability and proliferation with nanosilver treatment, Castiglioni et al. [148] reported that non-toxic 35 nm nanosilver treatment of human microvascular endothelial cells (HMEC) decreased the cellular proliferation while not effecting cellular viability. This inhibition did not permanently affect the cells since cell proliferation increased again after the removal of the nanosilver [148]. Female Wistar rats treated with an intraperitoneal injection of 50–60 nm nanosilver resulted in decreased angiogenesis in the ovarian tissue visualized via immunofluorescence [149]. In a wound healing study using NHDF cells and NHEK cells, decreased production of VEGF was seen for both low and high treatment concentrations of 10 nm nanosilver, indicating decreased angiogenesis, with a greater inhibition of VEGF production seen in NHEK cells [90]. Wounds on male Wistar rats healed more rapidly with treatment of non-toxic doses of 10–20 nm nanosilver, followed by rats treated with toxic doses of nanosilver, the positive control rats treated with silver alginate cream, and finally the negative control rats with no treatment healing last. Histological analysis of the wounds revealed decreased angiogenesis in the wounds for the rats treated with the non-toxic nanosilver dose. Thus non-toxic nanosilver treatment of wounds was found to aid in healing [98,99].

Anti-angiogenesis effects of nanosilver have also been reported using in vivo angiogenesis models such as the chick chorioallantoic membrane assay (CAM), Matrigel implant, and aortic ring models. Decreased angiogenesis in the mouse matrigel model and the CAM model was observed with nanosilver (coated with diaminopyridinyl (DAP)-derivatized heparin (HP) polysaccharide (DAPHP); 10–30 nm) when angiogenesis was induced using FGF-2 [150]. Male mice (C57BL/6NCr) subcutaneously injected with Matrigel containing FGF-2 and nanosilver for 12 days had decreased hemoglobin content in the Matrigel with the nanosilver as compared to the Matrigel with just FGF-2, indicating decreased angiogenesis. Additionally in the CAM model, the number of FGF-2 induced new blood vessel branch points was seen to decrease with co-treatment of the nanosilver [150]. In another study, treatment with 16.5 nm nanosilver resulted in decreased blood vessel formation, decreased hemoglobin content, and smaller chick embryos in the CAM assay [151]. Treatment with 12 nm nanosilver resulted in smaller and fewer blood vessels formed in a rat aortic ring model [152]. Interestingly, treatment with larger 100 nm nanosilver did not significantly inhibit angiogenesis in the CAM assay, showing the importance that the size of the nanoparticles has on the effect [153].

The only report of nanosilver increasing angiogenesis is by Kang et al. [154], who found increased blood vessel formation and hemoglobin content with the Matrigel assay in female C57BL/6 mice. In this study, angiogenesis was not induced with growth factor treatment, and the Matrigel was only mixed with the 2.3 nm PVP–coated nanosilver. After 10 days, the Matrigel implants had increased hemoglobin content compared to the controls [154].

In addition to activating PI3K, VEGFR also phosphorylated focal adhesion kinase (FAK), ER retention defective 1 (ERD1), ER retention defective 2 (ERD2), p38 mitogen-activated protein kinase, and endothelial nitric-oxide synthase (eNOS) [154]. SVEC4-10 mouse endothelial cells had increased levels of VEGF and nitric oxide with the nanosilver treatment; as well as increased levels of phosphorylated and active FAK, AKT, ERK1/2, and p38, indicating increased VEGFR signalling [154]. Some of the key differences between this study reporting increased angiogenesis and all the other studies reporting decreased angiogenesis are: angiogenesis was not induced by VEGF or FGF-2 along with the nanosilver treatment, a smaller size of nanosilver particles was used, and differences in the strain and sex of mice or type of cells used or different treatment doses and times. No cell viability assay data is included in this study, so the toxicity of the treatments cannot be determined or commented on. However, the treatment concentrations used were in the same range that has been used in other studies with different cell types. Thus, with the exception of the work by Kang et al. [154], the consensus of the studies to date is that nanosilver treatment inhibits angiogenesis.

## 10. Nanosilver and Epigenetics

Epigenetics refers to the heritable and reversible modifications that customize gene expression without involving mutation of the DNA sequence. These consist of DNA methylation, histone tail modifications, and post-transcriptional regulation by non-coding RNAs [155,156].

### 10.1. Nanosilver and DNA Methylation

DNA methylation in the promoter region of a gene generally represses transcription, while demethylation of the promoter region allows for transcription of the gene. Methylation may occur on adenine at the N-6 position in a 5′-G-A-T-C-3′ sequence or on cytosine at the C-5 position in a 5′-C-G-3′ (CpG) sequence. Methylation of the cytosine residues at CpG sites within the gene are also involved in gene regulation. Highly expressed genes have low promoter methylation and high gene-body methylation, while low expressed genes are methylated more in the promoter region and less in the body of the gene [157]. The methyl group is transferred from *S*-adenosylmethione (SAM) to the cytosine residue by DNA methyltransferases (DNMTs) [158]. DNA methylation on cytosine forms 5-methylcytosine (5-mc). This methylation inhibits the transcriptional machinery from accessing the DNA, as well as attracting methyl-CpG-binding domain (MBD) proteins which bind and further block transcription. DNA demethylation is not well understood, however, the transformation of 5-mc to 5-hydroxymethylcytosine (5-hmc) by the 10–11 translocation 1 (TET1) enzyme produces another important form of cytosine that plays a part in the demethylation process [155,156].

Blanco et al. [159] examined the effects of nanosilver on global DNA methylation using A549 cells, and found an increase in methylation after nanosilver (PVP-coated; 21.74 nm) treatment, however, the treatment used in this study was toxic to the cells. Increased amounts of 5-mc and of the DNMT enzymes were found in mouse hippocampal neuronal (HT22) cells after 48 h nanosilver (~8 nm) treatment at the EC_50_. Interestingly, elevated levels of 5-mc and DNMTs (especially DNMT2) were still seen even after the toxic nanosilver treatment was removed and the cells allowed to recover in media for an additional 96 h [160]. DNA methylation changes were also found on several genes extracted from the placentas of pregnant mice that had been treated intravenously with 8nm nanosilver. Specifically, methylation of the pleiomorphic adenoma gene-like 1 (*Zac1*) gene decreased, while methylation of the insulin-like growth factor 2 receptor (*Igf2r*) gene was marginally increased [7].

### 10.2. Nanosilver and Histone Tail Modifications

DNA is wrapped around histone proteins forming nucleosomes. Eight histone proteins form each nucleosome core—two each of H3, H4, H2A, and H2B—and these wrap approximately 147 base pairs of DNA [161]. The N-terminal tails of the histone proteins extend beyond the nucleosome core and are subject to various covalent modifications such as include acetylation, phosphorylation, methylation, ubiquitination, and SUMOylation, which determine how tightly the DNA is packed. Currently, studies examining the effects of nanosilver on histone tail modifications in cells have only looked at methylation, phosphorylation, and acetylation. Histone tail modifications and DNA methylation are also important in determining where the nucleosome is formed on the DNA, and thus greatly affect gene expression [22,155].

Methylation of lysine or arginine residues in the histone tails either relax or condense the DNA depending on the combination of residues that are methylated. For example, trimethylation of lysine 4 on H3 (H3K4me3) and monomethylation of lysine 79 on H3 (H3K79me1) allows for active gene transcription [162]. Non-toxic nanosilver treatment of MEL cells with 25 nm PVP-coated nanosilver (1 and 8 µg/mL) decreased the global methylation on histone 3 [22]. These erythroid precursor cells were differentiated into red blood cells by DMSO treatment, and the β-globin locus specifically examined. Western blot indicated that the overall levels of H3K4me3 and H3K79me1 were decreased in cells treated with nanosilver, while ChIP-PCR revealed that H3K4me3 and H3K79me1 decreased by at least 50% at the β-globin locus. The levels of histone methyltransferase enzymes: myeloid/lymphoid or mixed-lineage leukemia 2 (MLL2) involved in H3K4 trimethylation, and disruptor of telomere silencing 1 (Dot-1L) for H3K79 methylation, were also reduced. As well, pull-down and immunoprecipitation assays indicated that the nanosilver bound directly to the H3 and H4 histones. Not surprisingly, all this resulted in reduced RNA polymerase II activity at this locus, lower levels of β-globin mRNA and protein, and reduced amounts of hemoglobin produced in the cells [22].

Histone phosphorylation of serine 10 on H3 (p-H3S10) corresponds to condensation of the DNA [162]. Nanosilver treatment (≥100 nm, ~1 mg/mL) of MCF-7 cells, A549 cells, and human skin keratinocytes (HaCaT) increased the levels of p-H3S10 [162,163]. This occurred though nanosilver induced activation of Aurora kinases (AURKs) which regulates p-H3S10. Although more study is needed, Zhao et al. [163] suggests that the uptake of nanosilver allows for the release of silver ions into the cell, which then trigger the release of globular actin (G-actin) from filament actin (F-actin). This rearrangement of the cytoskeleton activates AURKs which phosphorylate H3S10 as part of the cellular response [162]. Phosphorylation of serine 139 on histone H2A.X (γ-H2A.X) is an indication of DNA damage due to double-strand breaks [164], and γ-H2A.X phosphorylation has been reported with both toxic and non-toxic nanosilver treatment in MCF-7 and A549 cells [76,159].

Histone tail lysine acetylation by histone acetyltransferases (HATs) opens DNA allowing increased expression, while deacetylation by histone deacetylases (HDACs) condenses the DNA, making it inaccessible for expression [156]. Deacetylation of H3 has been found to occur with toxic nanosilver (PVP-coated; 21.74 nm) treatment on A549 cells [159].

### 10.3. Nanosilver and Non-Coding RNA Regulation

Post-transcriptional regulation by non-coding RNAs such as microRNAs (miRNAs) and long non-coding RNAs (lncRNAs) are also involved in epigenetics [155,156]. MicroRNAs are short (22–25 nucleotides) single stranded non-coding RNAs that bind to target mRNA and either cause them to be degraded if there is complete complementarity between the miRNA and the mRNA, or cause the expression to be inhibited if there is incomplete complementarity [156]. Long non-coding RNAs are non-coding RNAs longer than 200 nucleotides. They serve to regulate miRNA action, mRNA gene expression, protein function, and cellular functioning [75].

Several studies have been done examining the effect of nanosilver exposure on post-transcriptional regulation by non-coding RNAs, with one study examining the effect on lncRNA regulation, and several studies examining the effect on miRNA regulation. The effect of nanosilver (PVP-coated; 11, 27, and 95 nm) on lncRNA regulation was examined using K562 cells [75]. Non-toxic nanosilver (≥100 nm) treatment significantly up- or downregulated several lncRNAs, with the lncRNA osteosarcoma doxorubicin-resistance related upregulated lncRNA (ODRUL) being upregulated the most by approximately 10-fold. A miRNA microarray with human Jurkat T cells clone E6-1 revealed that the expression of 19 miRNAs were upregulated and 44 miRNAs were downregulated [165]. Messenger RNA microarray data was compared, and an increase in metallothionein 1F (MT1F) and tribbles homolog 3 (TRIB3) mRNA corresponded with decreased expression of hsa-miR-219-5p miRNA. Network analysis of the interactions between the miR-219-5p miRNA and the MT1F and TRIB3 mRNA indicated that pathways involved in oxidative stress response, cell cycle, cell survival, and apoptosis would be affected [165]. Human embryonic stem cell-derived neural stem/progenitor cells (hESC-derived NPCs) are commonly used to study developmental neurotoxicity. In a study by Oh et al. [3], hESC-derived NPCs were treated with nanosilver (citrate-coated; 13.3 nm) and miRNA and mRNA microarrays were evaluated. At 6 h, the nanosilver treatment resulted in the upregulation of 11 miRNAs while 20 mi-RNAs were downregulated, together affecting the mRNA expression of 611 genes. At 24 h, the nanosilver was slightly toxic to the cells and 22 miRNAs were upregulated while 10 miRNAs were downregulated, together affecting the mRNA expression of 280 genes. The regulation of only 6 miRNAs and the mRNA expression of only 33 genes were the same for both treatment times. Analysis of the target genes affected by the changes in miRNA regulation due to the nanosilver treatment indicated that pathways involving Nrf2 mediated oxidative stress response were strongly affected at both treatment times. Neuronal function, neuronal signalling, and nervous system development were affected by the 6 h treatment, and the inflammation response pathway was affected by the 24 h treatment [3]. Treatment of human dermal fibroblasts (HDF) with non-toxic levels of nanosilver (20 nm) changed the regulation of 246 miRNAs and the expression of 25 proteins, affecting numerous cellular pathways and leading to actin cytoskeleton damage, a decrease in intracellular ATP, and activation of apoptosis [135].

In a different model system, the exposure of nematodes to nanosilver (PVP-coated; 58.3 nm) at the EC_30_ for 10 generations resulted in an approximately 10-fold sensitization that did not dissipate in the subsequent generations that were also exposed to nanosilver [27]. Nematodes exposed to nanosilver for only five generations retained the sensitivity in later generations even though they were no longer exposed to the nanosilver, indicating epigenetic effects [27]. Thus, epigenetic changes in terms of increased DNA methylation, various histone tail modifications, and changes in non-coding RNA expression were seen with nanosilver treatment.

## 11. Nanosilver and Genotoxicity

Genotoxicity refers to damage to a cell’s DNA or RNA by a genotoxic agent [166,167]. This DNA damage could include mutations, DNA adduct formation, oxidation of the DNA bases, single or double strand breaks, the formation of crosslinks, and structural changes. Genotoxic nanoparticles can induce DNA damage either directly through DNA binding; or indirectly through binding to associated proteins, the mitotic spindle components, or producing oxidative stress [168]. The main effects that need to be evaluated in order to determine genotoxicity are the presence of DNA damage, structural and numerical chromosomal aberrations, and mutations. Some DNA and RNA damage may be able to be repaired by the cellular repair mechanisms. However, chromosomal aberrations and mutations are irreversible and may lead to cancer, or to heritable diseases if the damage occurs in the germ cells [166,167,169]. The majority of studies, both in vitro and in vivo, indicate that nanosilver has genotoxic effects; however, other studies do not report any genotoxicity.

### 11.1. Nanosilver and Genotoxicity In Vitro Studies

Many in vitro genotoxicity assays may be done, however, these in vitro assays tend to be highly sensitive but not very specific and may give false positive results making follow up in vivo testing necessary for confirmation [169]. Common in vitro genotoxicity tests include the Ames test for mutagenicity in bacteria, the alkaline single-cell microgel electrophoresis (comet) assay for DNA damage, the in vitro mammalian chromosomal aberration test, the in vitro mammalian cell micronucleus test, and the in vitro mammalian cell gene mutation test (thymidine kinase (TK) mouse lymphoma assay (MLA), or hypoxanthine phosphoribosyl transferase (HPRT) gene mutation assays) [167,170]. The comet assay detects DNA single and double strand breaks, incomplete excision repair sites, alkali labile sites, and DNA base oxidation [20,57,81,168]. Histone H2A.X phosphorylation on serine 139 is an early indication of DNA double strand breaks [164]. DNA strand breaks can also be detected using Hoechst staining [84]. The presence of DNA adducts can be detected with ^32^P-postlabeling, and the levels of 8-oxoguanine may be used as an indication of the levels of DNA base oxidation since 8-oxoguanine is the main purine oxidation product [168], which, if not repaired, may lead to the formation of transversion mutations [51]. Non-toxic nanosilver (46 nm) treatment of hMSC cells resulted in the presence of nanosilver in the nucleus and caused DNA damage. The comet assay as well as the chromosome aberration test indicated genotoxicity, and the chromosome aberrations were found to consist of both chromatid deletions and chromatid exchanges [57]. DNA damage was detected with the comet assay in MCF-7 cells, and the comet tail length was significantly increased in cells treated with non-toxic nanosilver (10–30 nm) above the EC_50_ level [81]. Interestingly, the genotoxicity observed by the comet assay in HMEC cells treated with 35 nm nanosilver was not permanent, and was no longer observed once the treatment was stopped and the cells were allowed time to recover [148]. Nanosilver (BSA-coated; 15.9 nm) treatment induced an increase in the number of DNA adducts in CHO-K1 cells at low treatment concentrations, while higher treatment concentrations were needed in order to observe an increase in 8-oxoguanine levels and micronuclei formation [64]. An increase in 8-oxoguanine in human Chang liver cells treated with nanosilver (5–10 nm) was accompanied by a decrease in both the gene and protein expression of 8-oxoguanine DNA glycosylase 1 (OGG1), a base excision repair enzyme that excises and repairs 8-oxoguanine [171]. DNA damage via the comet assay and micronuclei formation were observed in U251 and IMR-90 cells treated with starch-coated 6–20 nm nanosilver [62]; as well as in Chinese hamster ovary cells treated with ≥100 nm nanosilver [172]. HepG2 cells treated with non-toxic concentrations of nanosilver (polyethylenimine-coated; 7–10 nm) resulted in micronuclei formation in the cells [53]. In order to try to separate the effects due to the nanosilver particle itself and the Ag^+^ ions, cysteine was added to bind the Ag^+^ ions. This inhibited cell death and reduced, but did not eliminate, the micronuclei formation in the cells, indicating that both the nanosilver and the Ag^+^ ions affected it [53].

DNA microarray data indicated the upregulation of several stress responsive genes, such as for MT and heat shock proteins, as well as for genes involved in DNA repair such as the RAD 51 family member, *RAD51C* [53]. Similar results are reported by Sahu et al. [58] in a DNA microarray study with HepG2 cells treated with non-toxic nanosilver (20 and 50 nm) concentrations, with genes for MTs, heat shock proteins, and DNA repair (such as growth arrest and DNA damage-inducible 45 (GADD45)) being upregulated [58]. The protein expression of RAD51 also increased in TM3 cells and TM4 cells treated with nanosilver (10 and 20 nm) at the EC_50_ level [68,173]. An increase in γ-H2A.X histone phosphorylation at serine 139 was found with non-toxic nanosilver treatment (≥100 nm) in MCF-7 cells and was thought to be the result of double strand breaks from the nanosilver treatment due to the time needed before the phosphorylation occurred [76]. Similarly, an increased level of γ-H2A.X phosphorylation was seen in A549 cells with both toxic and non-toxic treatments PVP-coated nanosilver (21.74 nm) [159]. DNA damage due to double strand breaks was also indicated in HepG2 and Caco-2 cells using Hoechst 33258 staining [84].

No increase in genotoxicity was observed in human skin keratinocyte (HaCaT) cells treated with non-toxic levels of 30 nm citrate-coated nanosilver and tested with the comet assay [174]. As well, mutagenicity was not seen in an assay involving mouse embryonic fibroblasts (MEF-*LacZ*) containing the bacterial *lacZ* gene and treated with 20, 80, and 110 nm nanosilver [175]. The Ames test has been found to generally result in negative results when testing various nanoparticles [176], and was also found to be negative for mutagenicity in *Salmonella typhimurium* bacterial strains treated with nanosilver (≥100 nm) [172]. Additionally, no genotoxicity was found in ToxTracker mouse embryonic stem cells containing a DNA damage and replication stress related Berardinelli-Seip congenital lipodystrophy 2 (BSCL2)-GFP reporter or with the comet assay when treated with non-toxic levels of nanosilver (10 and 40 nm; citrate-coated) [20].

### 11.2. Nanosilver and Genotoxicity In Vivo Studies

Several in vivo studies report genotoxicity due to nanosilver at extremely high treatment concentrations. Examining the genotoxicity of nanosilver with different coatings, Nallanthighal et al. [51] treated male and female C57BL/6J *p^un^*/*p^un^* mice orally with 20 nm citrate- or PVP-coated nanosilver (4 mg/kg) for seven days. This dose is 800 times the daily oral exposure amount in humans that is thought to be safe by the United States Environmental Protection Agency (EPA), and is in the range of Ag exposure that could cause argyria. The citrate-coated nanosilver at this dose induced DNA damage in vivo, whereas the PVP-coated nanosilver and the Ag^+^ ion control did not. Chromosomal aberrations in the form of micronuclei were detected in the bone marrow. As well, increased 8-oxoguanine indicating DNA oxidation, and phosphorylation of histone H2A.X indicating the presence of double strand breaks were detected in the peripheral blood leukocytes of mice treated with citrate-coated nanosilver [51]. Patlolla et al. [177] observed increased micronuclei formation, as well as structural chromosome aberrations, DNA damage as seen via the comet assay, and a decrease in cell division measured by the mitotic index in bone marrow cells of male Sprague Dawley rats treated orally with 10 nm nanosilver (5, 25, 50, 100 mg/kg) once a day for five days. The higher doses (50 and 100 mg/kg) induced a significant increase in all of the endpoints tested, and resulted in an increase in ROS as measured by H_2_DCFDA [177]. Not surprisingly, the treatment of mice deficient in the base excision repair enzyme OGG1 results in increased genotoxicity in the mice [178].

In contrast to the above studies, other studies do not find genotoxicity. In a study by Kim et al. [37], male and female Sprague Dawley rats were treated orally with nanosilver (60 nm) for 28 days at0, 30, 300, and 1000 mg/kg. The quantity of micronucleated polychromatic erythrocytes and the ratio of polychromatic erythrocytes to total erythrocytes did not change in the nanosilver treated rats; indicating no evidence of DNA damage and no genotoxicity to the bone marrow cells, respectively. However, there was some evidence of slight liver damage in the rats treated with the highest nanosilver treatment level, as the alkaline phosphatase and cholesterol levels were affected in these rats [37]. In another in vivo study by Kim et al. [179], using a longer treatment time and inhalation instead of oral exposure, male and female Sprague Dawley rats were exposed to nanosilver (18 nm) by inhalation for 90 days at 0.7 × 10^6^, 1.4 × 10^6^, and 2.9 × 10^6^ particles/cm^3^ exposure amounts. Once again, there was no change observed in the frequency of micronucleated polychromatic erythrocytes, and the ratio of polychromatic erythrocytes to total erythrocytes did not change, indicating no genotoxic affects in the rat bone marrow [179]. Wen et al. [180] also found no increase in bone marrow micronuclei in female Sprague Dawley rats treated with nanosilver (6.3–629 nm, 5 mg/kg) intravenously for 24 h. However, there was a 14.3% increase in aberration cells, a 7.1% increase in multiple aberration cells, and a 4.3% increase in the number of polyploidy cells in the nanosilver treated rats. Treatment with Ag^+^ ions (0.0003 mg/kg) resulted in a higher increase in aberration cells and multiple aberration cells, but only 0.1% polyploidy cells, indicating either that this might be a nanosilver specific effect or that the low amount of polyploidy cells in the Ag^+^ ion treated rats may be due to chromosome fragmentation [180].

## 12. Nanosilver and Cancer

Cancer in its various forms is a devastating disease, and results from a combination of environmental, physiological, and genetic factors [16]. Cancer cells are resistant to pro-death and anti-proliferative signals, are self-sufficient in terms of growth signals, and can replicate limitlessly. In order to form and grow, it is thought that cancer must first be initiated in a cell through impaired mitochondrial function or through various genetic mutations and rearrangements, resulting in irreversible changes to the cell, activated oncogenes, and dysfunctional tumor suppressor proteins [89,181,182]. Growth of the cancer cell must be promoted by the surrounding cellular environment, and if not checked by the immune system, the disease can then progress with increased tumor growth and metastasis [89]. Many current cancer treatments have only limited effectiveness, and also produce undesired and damaging side effects. Thus, new treatments, combinations of treatments, and the incorporation of nanoparticles such as nanosilver into cancer treatments are currently being examined [16,183].

### 12.1. Response of Cancer vs. Non-Cancer Cells to Nanosilver Treatment

Cancer cells of various cell lines have been found to be more susceptible to nanosilver treatment than non-cancer cells. The relative sensitivities of non-cancer and cancer cells to nanosilver was examined using primary mouse embryonic fibroblast (P-MEF) cells and immortalized mouse embryonic fibroblast (I-MEF) cells respectively, and both cell types were treated with the same nanosilver treatment (20 μg/mL for 24 h). The cancer-like I-MEF cells were much more susceptible and had a drastically reduced cell viability, with only 28.03% viable cells compared to the non-cancerous cells which remained 91.56% viable [18]. A549, human ovarian cancer cells (2780), human breast adenocarcinoma cells (MCF-7), and human breast adenocarcinoma cells (MDA-MB 231) all demonstrated a significant decrease in cell viability when treated with 0–50 μg/mL nanosilver (10 nm); whereas normal lung epithelial cells (L132) remained completely viable in that treatment range [83]. In human breast cancer and non-cancer cells, nanosilver (2 and 15 nm) treatment produced increased cell death in the cancerous MCF-7 and T-47D cells than in the non-cancerous MCF-10A cells [117]. Interestingly, nanosilver (starch-capped, 6–20 nm) induced DNA damage to a much greater extent in cancerous human glioblastoma (U251) cells than in non-cancerous IMR-90 cells [62]. Nanosilver (23.44 nm, PVP-coated) was more cytotoxic to HepG2 cells than to normal hepatic L02 cells in terms of decreased mitochondrial membrane potential and cell membrane leakage. Additionally, the nanosilver induced an increase in ROS as measured with H_2_DCFDA in the cancerous HepG2 cells, but not in the non-cancerous L02 cells, and triggered mitochondrial-mediated apoptosis and cell death via the Fas death receptor pathway in the HepG2 cells [184].

### 12.2. Mechanisms Involved in the Effect of Nanosilver on Cancer Cells In Vitro

Numerous studies report the anti-cancer effects of nanosilver treatment on various cancer cells in vitro [120,153,185,186,187,188,189,190], with many pathways being involved. The expression of HIF-1α aids in cell survival under low oxygen conditions and is found to be high in cancer cells inside solid tumors. The high level of HIF-1α aids the cancer cells in their resistance against treatment and death. This was demonstrated in A549 cells with biosynthesized nanosilver (10 nm) treatment at the EC_50_ level, where pre-exposure to hypoxia in order to induce the expression of HIF-1α attenuated the nanosilver induced cell death [83]. Nanosilver has been found to work against this survival mechanism in cancerous MCF-7 cells, and treatment with nanosilver (10 nm) was able to significantly inhibit the production of HIF-1α through decreasing activation of the hypoxic response element, even when this pathway was stimulated by hypoxia or by the hypoxic mimic cobalt chloride (CoCl_2_) [60]. This decreased the protein levels of HIF-1α and HIF-2α present in the cells, reducing the expression of HIF-1α target genes such as VEGF-A and GLUT1, and serving as both an anti-cancer and anti-angiogenesis agent [60]. This is important since cancer cells stimulate increased angiogenesis in order to bring in more oxygen and nutrients, enabling the cancer to grow and spread [140,142]. Cancerous human pancreas ductal adenocarcinoma (PANC-1) cells were approximately two times more susceptible to nanosilver induced cell death than non-cancerous immortalized human pancreas duct epithelial (hTERT-HPNE) cells when treated with either 2.6 nm or 18 nm nanosilver; with the smaller 2.6 nm nanosilver being more toxic to both cell lines than the larger 18 nm nanosilver [54]. Early apoptosis was mainly induced in the cancerous PANC-1 cells with nanosilver concentrations up to the EC_50_ concentration, above which late apoptosis and necroptosis began increasing. In PANC-1 cells, nanosilver increased the protein expression of LCS-II, potentially indicating the induction of autophagy. Necroptosis was also induced with an increase in protein expression of receptor-interacting serine/threonine-protein kinase 1 (RIP1), receptor-interacting serine/threonine-protein kinase 3 (RIP3), and mixed lineage kinase domain-like pseudokinase (MLKL). The pro-apoptotic protein BAX increased in expression, while the anti-apoptotic protein BCL2 decreased in expression and the protein expression of the tumor suppressor transcription factor, p53, also increased [54]. This transcription factor is involved in inducing cell cycle arrest, apoptosis, and cell senescence [72]. Activated p53 induces cell cycle arrest, and one of the downstream genes activated by p53 is p21, a cyclin kinase inhibitor (CKI) involved in cell cycle arrest at the G1 phase [191]. This arrest allows for DNA repair to occur, thus preventing the replication of damaged DNA. However, if the DNA damage is too great and repair is not feasible, apoptosis is triggered [58,74]. HepG2 cells treated with a non-toxic concentration of nanosilver (20 nm), analysis of DNA microarray data revealed that many genes involved in the cancer pathway were upregulated. As well, TGF-β, MAPK, and the p53 signalling pathways were all upregulated and formed an intracellular signalling cascade [58]. In fact, p53 signalling was found to be essential for the mitochondrial mediated apoptosis via the MAPK signalling cascade that was triggered by high nanosilver (20 nm) treatment in HCT116 cells [120].

Buttacavoli et al. [16] conducted an integrated proteomic study exploring the biochemical mechanisms in cancer cells affected by treatment with biosynthesized 11 nm nanosilver embedded in a specific polysaccharide (extracellular polymeric substance (EPS)) via the bacteria *Klebsiella oxytoca* DSM 29614. The biosynthesized nanosilver was tested on human breast cancer cells SKBR3 and 8701-BC, and human colon cancer cells HT-29, HCT 116, and Caco-2, and found to be cytotoxic to all of the cancer cells tested, with the SKBR3 cells being the most susceptible. Some of the treated SKBR3 cells underwent apoptosis (11%), and evidence of activated autophagy was observed. The cytotoxicity of the nanosilver on the SKBR3 cells was compared to the effect on non-cancerous HB2 mammary epithelial cells, with the nanosilver treatment being selectively toxic to the cancer cells and having a selectivity index similar to the commonly used chemotherapy drug doxorubicin. Nanosilver treatment of the SKBR3 cells at both a non-toxic concentration and at the EC_50_ concentration greatly decreased the cell motility and colony forming capacity of the cancer cells. MMP enzymes are important in the process of cell migration, and the activity and protein levels of MMP-2 and MMP-9 decreased with EC_50_ nanosilver treatment. Differential gel electrophoresis (2D-DIGE), proteomic identification, and analysis using the Search Tool for the Retrieval of Interacting Genes/Proteins (STRING) database for protein-protein interactions and the Database for Annotation, Visualization and Integrated Discovery (DAVID) bioinformatics resource for pathway identification, together indicated that the nanosilver treatment mainly affected the expression of proteins involved in pathways in the mitochondria, ER, oxidative stress response, apoptosis regulation, and downregulated enzymes involved in glycolysis [16]. A proteomics study by Verano-Braga et al. [43] using human colon carcinoma (LoVo) cells found that exposure to 20 nm citrate-coated nanosilver induced protein carbonylation and ROS as measured with H_2_DCFDA. The proteomics results were analyzed with gene ontology and STRING and were corrected for effects by released Ag^+^ ions in order to examine the nanosilver particle effect only. Proteins involved in the proteasome, translation initiation, oxidative stress response, and cell death were upregulated. Mitochondrial electron transport chain proteins decreased, either through downregulation or degradation, as did proteins involved in cell growth, spliceosome function, and mitochondrial translation. Specifically, small ubiquitin-related modifier 2 (SUMO2), a member of the SUMO protein family, and tripartite motif containing 28 (TRIM28), an E3 ligase involved in SUMOylation, were both upregulated by the nanosilver treatment, indicating activation of the SUMO pathway. However, the SUMOylation protein targets could not be identified in this study [43].

### 12.3. Cancer In Vivo Studies with Nanosilver Treatment

In a study by Sriram et al. [192], Dalton’s lymphoma ascites (DLA) were used to produce tumors in female Swiss albino mice. Then starting the following day, the mice were treated with 500 nM of 50 nm nanosilver via intraperitoneal injection for 15 days, with the treatment concentration of 500 nM being the EC_50_ for DLA cells. Not surprisingly, this high of a treatment concentration activated caspase-3, induced DNA fragmentation, and lead to apoptosis in the DLA cells. However, in the non-cancerous control mice that were treated with 500 nM nanosilver there were no signs of toxicity such as appetite loss, reduction in body weight, fatigue, or fur color change that were seen. The tumor-bearing mice were greatly helped by the nanosilver treatment. They had significantly fewer malignant DLA cells in their peritoneal fluid and they lived for 50% longer than the untreated tumor-bearing mice. As well, the nanosilver treatment decreased the excess ascetic fluid by 65%, bringing the body weight and white blood cell and platelet counts in the ascetic fluid back to normal. Thus in this study, nanosilver treatment successfully inhibited cancer growth without causing adverse effects in the mice [192]. Male Swiss albino mice with tumors formed from DLA cells were treated intraperitoneally with nanosilver (35 µg/kg body weight) for 10 days following the injection of the cancer cells, and this treatment was found to have a similar effectiveness to treatment with the anti-cancer drug 5-Fluorouracil (20 µg/kg body weight) [193]. The nanosilver treated mice lived 46.52% longer than the mice with no treatment and experienced reduced tumor volume. Additionally, the increase in angiogenesis observed in the non-treated tumors was successfully controlled with the nanosilver treatment. The cancer induced decrease of SOD and GSH levels in the liver, and increase in the level of the lipid peroxidation product, MDA, all returned to normal with the treatments. Liver damage and leakage of the liver enzymes glutamate oxalate transaminase (SGOT), glutamate pyrubate transaminase (SGPT), and alkaline phosphatase (ALP) into the blood serum was stopped. Cancer induced hypercalcemia and elevated LDH levels were also corrected. Gene analysis of the cancer cells in the tumors revealed that the nanosilver treatment induced apoptosis in the cells through both the intrinsic mitochondrial pathway and through the extrinsic death receptor pathway, with the gene expression of p53, cytochrome C, caspase-3, caspase-8, caspase-9, and caspase-12 all being upregulated [193]. One-time nanosilver (citrate-coated, 5 nm) treatment via peritumoral injection around tumors made from murine lung squamous tumor cells (KLN 205) in female immune competent DBA/2 mice and immune deficient NOD SCIDγ mice initially reduced the size of the tumors in both types of mice [91]. After this treatment, the effects of the nanosilver gradually dissipated in the tumors in the immune deficient NOD SCIDγ mice, and the tumor growth rate gradually increased back to the initial growth rate. The growth rate of the tumors in the immune competent DBA/2 mice was greatly reduced after the nanosilver treatment and did not recover, possibly due to the immune response triggered by the nanosilver treatment in these mice [91].

Nanosilver modified with the cell penetrating TAT peptide to improve cellular uptake has been found to be an effective and promising anti-cancer treatment against both multidrug-resistant (MDR) cancer cells (MDR melanoma B16 cells and MDR breast cancer cells MCF-7/ADR) and non-resistant cancer cells (HeLa and MCF-7 cells), [194]. Both the TAT-modified nanosilver (8 nm) and the un-modified PVP-coated nanosilver were effective in inhibiting cancer cell proliferation, with the TAT modified nanosilver being 4, 7, 24, and 9 times more potent than the un-modified nanosilver on the B16, MCF-7/ADR, HeLa, and MCF-7 cells respectively. Female nude mice with MDR B16 melanoma tumors simulating late stage MDR cancer exhibited inhibition of tumor growth with nanosilver and TAT-modified nanosilver peritumoral treatment, with the TAT-modified nanosilver being as effective as the doxorubicin (DOX) treatment (>85% growth inhibition) but without the debilitating adverse effects that generally makes DOX treatment unfeasible [194].

The levels of p53, p21, and cleaved caspase-3 increased in the liver tissue from male Sprague Dawley rats treated orally with up to 100 mg/kg/day PVP-coated nanosilver (20–30 nm) for 90 days, and then decreased again at higher treatment where autophagic cell death was thought to occur [74].

### 12.4. Nanosilver and Radiation Treatment

Nanosilver treatment (PVP-coated, 15.47 nm) of human U251 glioblastoma cells was found to increase cell death when used in combination with radiation treatment, and this cell death further increased when additionally combined with the autophagy inhibitor, 3-MA [45]. In HepG2 cells, nanosilver treatment increased the effectiveness of radiation in inducing apoptosis in HepG2 cells, with an upregulation in the expression of caspase-3 and BAX, a downregulation in the expression of BCL2, induced DNA damage, and a decrease in the levels of CAT, SOD, and total GSH [195]. Similarly, citrate-coated nanosilver (15.26 nm) was successful in inducing cell death in U251 cells and sensitizing the cells to radiation treatment [66]. In vivo experiments using female BALB/c nude mice with U251 glioma tumors indicated that the mice treated with nanosilver via intratumoral injection lived longer than the control mice, followed by the mice treated with radiation alone, and finally by the mice treated with a combination of radiation and nanosilver who survived for the longest length of time [66].

### 12.5. Nanosilver in Combination with other Drug Treatments

Lin Huang et al. [18] reported that nanosilver treatment in combination with autophagy inhibition increased the cell death and apoptosis seen in HeLa cells with nanosilver treatment, and that the autophagy induced by nanosilver treatment aided the HeLa cells to survive. This was further tested in mice by subcutaneously injecting mouse B16 melanoma cells into male C57BL/6 mice. After five days to allow the tumor to establish, the mice were treated with saline, wortmannin (an autophagy inhibitor), nanosilver, or nanosilver with wortmannin via subcutaneous injection into the tumor once a day for eight days. Wortmannin treatment by itself did not have any effect on tumor growth, however, nanosilver treatment decreased the weight of the tumor by 42.10%, and nanosilver with wortmannin treatment decreased the tumor weight by 60.91%. To confirm the increased cell death, the tumor tissue was examined with the TUNEL assay, which indicated that the tumors treated with wortmannin and nanosilver had the most apoptotic cells followed by the tumors treated with nanosilver alone [18]. Treatment of A2780 cells with a combination of nanosilver (18 nm) and salinomycin, a monocarboxylic ionophore effective against the growth of cancer stem cells, both at their EC_25_ concentrations, significantly increased cell death to 81% [72]. The proteins p53 and p21 were both upregulated by nanosilver treatment, and a combination of nanosilver plus salinomycin had an additive effect. The TUNEL assay indicated a significant increase in DNA fragmentation with the nanosilver and salinomycin treatments, and especially with the combined treatment [72]. A nanocomposite made with nanosilver dispersed on graphene sheets had greater toxicity than just the nanosilver in A2780 cells and HeLa cells, and co-treatment of the cancer cells with the common chemotherapy drug cisplatin and the nanosilver-nanocomposite further increased toxicity in the cells [183,185].

## 13. Interactions with, or Effects on, other Pathways

### 13.1. Nanosilver and the Cell Cycle

DNA damage in cells can disrupt the cell cycle causing an accumulation of cells halted in one of the cell cycle phases: gap 1 (G1), DNA synthesis (S), or the gap 2 (G2)/mitosis (M) phase. An accumulation of cells in the subG1 phase is indicative of apoptotic cells, potentially due to irreversible DNA damage. Nanosilver (starch-capped, 6–20 nm) treatment resulted in cell cycle arrest in the G2/M phase, with cancerous human glioblastoma (U251) cells being more affected than non-cancerous IMR-90 cells [62]. Cell cycle arrest also occurred in the G2/M phase in HK-2 cells treated with higher nanosilver (7.5 nm) treatment concentrations which appeared to result in less than 70% cell viability [82]. Cyclin-dependent kinase 1 (CDK1, also called cell division cycle protein 2 homolog (CDC2)) is important for cell cycle progression from the G2 to the M phase, and with nanosilver treatment more CDC2 was in its phosphorylated and inactive form. The enzyme responsible for activating CDC2, CDC25, was itself also more phosphorylated and inactive. As well, the protein expression levels of G2/mitotic-specific cyclin-B1 (cycline B1) were also decreased. The protein expression levels of p53 and p21 were both increased, which are involved in cell cycle arrest due to DNA damage, and the presence of DNA damage was confirmed with the cytokinesis-block micronucleus (CBMN) assay. These effects were increased by Nrf2 knockdown. Pretreatment with the antioxidant NAC alleviated the observed cell cycle arrest, DNA damage, increase in ROS; while pretreatment with l-buthionine-[*S*,*R*]-sulfoximine (BSO), which inhibits the synthesis of GSH, exacerbated the effects. Together, this exemplified the importance of the antioxidant response involving Nrf2 in protecting the cells from the DNA damage and cell cycle arrest induced by nanosilver treatment [82]. Treatment of human embryonic stem cell-derived neural stem/progenitor cells (hESC-derived NPCs) with 13.3 nm citrate-coated nanosilver resulted in an increase in apoptotic cells in the sub-G1 phase with both non-toxic, as well as toxic, nanosilver treatment. Increased DNA fragmentation was also seen using an ELISA assay confirming apoptotic cell death [3].

### 13.2. Effects of Nanosilver on DNA Polymerase and Transcription

Wang et al. [13] found that nanosilver can bind directly to DNA polymerase and decrease its activity in MEL cells. These erythroid progenitor cells highly express hemoglobin under normal circumstances, however, non-toxic nanosilver treatment with both spherical (10, 25, 40, and 110 nm) and plate-like (45 nm) PVP-coated nanosilver resulted in decreased mRNA expression of both α-globin and β-globin. Interestingly, the intracellular iron levels which are vital for hemoglobin production were not affected by the nanosilver treatment, and thus was not the reason for the decrease. Non-toxic nanosilver treatment (PVP-coated, 25 nm) caused silver to be accumulated in the nucleus of the MEL cells, directly interacted with RNA polymerase but not with the DNA as seen via pull down and immunoprecipitation assays, decreased RNA polymerase transcription, and decreased the amount of total RNA synthesized by over 30%. This same mechanism of suppressed RNA polymerase activity and suppressed transcription also occurs in *E. coli* cells treated with non-lethal concentrations of nanosilver and contributes to the antibacterial activity that is caused by the nanosilver. In both the MEL and *E. coli* cells, this mechanism is not a result of the released Ag^+^ ions from the nanosilver, but is a silver nanoparticle specific effect. The treatment of male and female BALB/c mice with nanosilver via intraperitoneal injection before pregnancy resulted in embryos with a pale and anemic appearance, reduced hemoglobin in the blood, and significantly reduced growth and development. Gene expression in the embryonic liver tissue was greatly affected with the upregulation of 37 genes and the downregulation of 264 genes, with many of the downregulated genes being involved in erythropoiesis [13]. Decreased transcription and RNA processing were also found with non-toxic nanosilver (9 nm) treatment in a study using *S. cerevisiae* gene deletion arrays as a high throughput screening technique to predict cellular effects [196].

### 13.3. Effects of Nanosilver on Pathways Involving Nrf2 and the Antioxidant Response

Nrf2 is a transcription factor that is integral to the antioxidant response and induces various xenobiotic-metabolizing and antioxidant enzymes through the electrophile response element/antioxidant response element (EpRE/ARE). When not stimulated, Nrf2 is bound to kelch-like ECH-associated protein 1 (Keap1) and Cullin-3 (Cul3) ubiquitin ligase in the cytosol where it is degraded through ubiquitination and subsequent proteasomal degradation. Nrf2 is activated as a protective response to various stressors such as ROS or electrophiles. Cysteine modifications on C151, C273, and C288 in Keap1 cause Nrf2 to be released and allow it to translocate to the nucleus where it binds to the antioxidant response element (ARE) along with small Maf proteins. This activates the expression of genes involved in the antioxidant response such as HO-1, NQO1, glutathione S transferases (GSTs), and many others [75,197,198]. Reporter gene assays for the Nrf2/ARE pathway have shown an increase in gene activation with nanosilver treatment. Prasad et al. [50] treated stable luciferase-reporter HepG2 cell lines for Nrf2/ARE, NF-κB, and AP1 with 10 and 75 nm, citrate and PVP-coated nanosilver, and found that the Nrf2/ARE pathway was activated the strongest out of the pathways tested [50]. N27 neurons transfected with Nrf2/ARE reporter gene also resulted in activation of the Nrf2/ARE pathway with nanosilver (10 and 75 nm, PVP-coated) treatment, and the expression of oxidative stress related genes HO-1 and NQO1 also increased [69]. DNA microarray data indicated that genes involved in the Nrf2 oxidative stress response pathway were upregulated in HepG2 cells treated with non-toxic nanosilver (20 and 50 nm) treatments [58]. Oxidative stress was induced and the protein levels of Nrf2 and HO-1 were greatly elevated in K562 cells when treated with non-toxic levels of 27 nm PVP-coated nanosilver [75]. The upregulation of the lncRNA ODRUL as a response to nanosilver exposure is potentially controlled by Nrf2, since pre-treatment with the antioxidant NAC decreased the nanosilver induced ODRUL upregulation by more than half, and Nrf2 knockdown completely inhibited the ODRUL upregulation in the K562 cells. Once upregulated by the nanosilver treatment, ODRUL physically interacts with phosphatidylinositol 4-kinase alpha (PI4Kα), as was seen through RNA-protein pull down experiments, and regulates the subsequent PI4Kα, AKT, JNK, and BCL2 signalling [75]. On the other hand, in human Chang liver cells, nanosilver (5–10 nm) treatment resulted in decreased protein expression of phosphorylated and active AKT and ERK1/2. AKT and ERK are both involved in the regulation of Nrf2, and decreased AKT and ERK resulted in decreased Nrf2 protein expression in this study. With nanosilver treatment, the decreased Nrf2 did not bind to the OGG1 gene promoter, the expression of OGG1 was decreased, and thus the levels of the DNA oxidation marker, 8-oxoguanine, were not repaired and increased in the cells [171].

### 13.4. Nanosilver and the Insulin Signalling Pathway

In the insulin signalling pathway, insulin binds to the insulin receptor tyrosine kinase, which activates insulin receptor substrate 1 (IRS-1) by phosphorylation, leading to activation of AKT and mTOR. In the liver, AKT regulates glucose metabolism and is involved in inducing glycogen synthesis via the phosphorylation and inhibition of glycogen synthase kinase-3 beta (GSK3β). The levels of phosphorylated AKT increased in the livers of male Sprague Dawley rats treated orally with up to 100 mg/kg/day nanosilver (PVP-coated, 20–30 nm), as did the levels of phosphorylated IRS-1, phosphorylated GSK3β, and phosphorylated mTOR [74].

### 13.5. Nanosilver Effects on Copper Homeodynamics

Copper is an essential micronutrient in the human body. Whether nanosilver has any effects on copper homeostasis in human cells or in the human body is currently unexplored. However, non-toxic nanosilver treatment of *Drosophila melanogaster* with citrate-coated nanosilver (1–50 nm) has been found to result in cuticular demelanization and to detrimentally effected their ability to move, a symptom that is also seen with copper deficiency [21]. Additionally, the activities of copper dependent enzymes such as tyrosinase and copper/zinc superoxide dismutase (Cu/ZnSOD) were decreased, with tyrosinase being involved in melanin synthesis. The activity of manganese dependent SOD (MnSOD) was not affected, indicating a specific effect on intracellular copper levels. Nanosilver was also found to interact with the copper transporters (CTR). Thus, the nanosilver appears to competitively compete with the copper ions for uptake by the transporters, resulting in a lower amount of copper being brought into the cell and cellular copper depletion [21,24]. A related study done by Ilyechova et al. [40] examined the effects of Ag^+^ ions on albino rats, using AgCl mixed into the food. Copper deficiency was observed in the rats, as well as decreased mRNA expression of several genes such as the cuproenzyme cytochrome c oxidase subunit 4 isoform 1 (Cox4i1), and genes involved in copper homeodynamics such as Cu(I)/Ag(I)-transporter 1 (CTR1), Cu(I)/Ag(I)-transporter 2 (CTR2), Cu-chaperone for SOD1 (CCS), MT1A, and Cu(II) binding cytosol protein (Commd1); although the protein expression of MT1A and Commd1 did not decrease [40].

### 13.6. Effects of Nanosilver on Brain Function

Amyloid protein aggregates formed from hen egg white lysozyme in the presence of a low concentration of nanosilver (108 nm), and injected into male Wistar rats as a model for Alzheimer’s Disease, disrupted the formation of amyloid aggregates, with the aggregates formed being non-toxic to the rats [199]. This also increased memory and spatial learning. Higher nanosilver concentrations had the opposite effect and increased amyloid formation, indicating an inverse dose-dependent inhibition [199]. The results of this study suggest that low dose nanosilver may be potentially beneficial in treating diseases involving protein misfolding and deposition such as Alzheimer’s Disease.

The effect of nanosilver treatment on the levels of various neurotransmitters were examined in female Wistar rats treated orally with nanosilver (PVP-coated; 14 nm) by gavage administration for 14 or 28 days [112]. After 28 days, the dopamine level increased when the rats were treated with 4.5 and 9 mg/kg body weight, while 5-hydroxytryptamine (5-HT) only increased when the rats were treated with 9 mg/kg body weight, and the noradrenaline levels were not affected. However, in a separate experiment, a shorter 14 day exposure at 2.25 and 4.5 mg/kg body weight resulted in a decrease in dopamine levels [112]. Thus, more studies need to be done in regards to the effects of nanosilver on neurotransmitter levels and brain function.

## 14. Conclusions

Nanosilver is generally taken up into cells through endocytosis, with the smaller sized nanosilver being taken up into the cells, interacting with the cellular components, and binding to biomolecules through their sulfhydryl groups. Larger sized nanosilver may be too large to be internalized and may remain on the outside where it can trigger various receptor-mediated signalling mechanisms or cause lipid peroxidation. Inside the cell, it has generally been accepted that nanosilver produces ROS and oxidative stress in the cells, although this has been questioned in some studies where no increase in ROS was seen. This may be due to different experimental conditions or due to problems detecting the actual levels ROS with H_2_DCFDA, the main dye that is used to detect ROS. Nanosilver activates the immune response and increases inflammation, with this having a beneficial effect and shortening the time required for wound healing. There are currently only a small number of studies that have been done on the effects of nanosilver on hypoxic stress, thus requiring additional research. High dose nanosilver treatment around the EC_50_ value for the cells leads to increased contact and signalling between the ER and the mitochondria, increased transfer of calcium from the ER to the mitochondria, mitochondrial dysfunction, decrease in ATP production, and mitochondria-mediated apoptosis. It has been found that nanosilver exposure can disrupt ER homeostasis and cause the induction of the ER stress response. The general consensus of the studies to date is that nanosilver treatment inhibits angiogenesis. Epigenetic changes in terms of increased DNA methylation, various histone tail modifications, and changes in non-coding RNA expression have been observed. In terms of genotoxicity, DNA damage, DNA base oxidation, DNA adducts, DNA strand breaks, and chromosomal aberrations have all been observed; however, not all studies observe this, and this effect may not be permanent once the nanosilver treatment is removed. Finally, cancer cells are consistently more susceptible to nanosilver treatment than non-cancer cells, and in vivo studies indicate that nanosilver is an effective and promising anti-cancer agent, with no adverse effects observed in the treated animals. This anti-cancer effect can be further improved by using the nanosilver treatments in combination with other cancer treatments.

Thus, in general, nanosilver in reasonable doses has many beneficial effects and applications, especially in its anti-bacterial, anti-viral, anti-fungal, anti-cancer, and wound healing functions; while at the same time not causing adverse effects in vivo. As with any compound, high enough doses will induce toxicity, especially in in vitro situations.
